# HAp/PLGA/Chitosan Scaffolds Fabricated by Freeze-Drying and 3D Printing for Bone Regeneration: In Vitro Evaluation and Finite Element Analysis

**DOI:** 10.3390/biomimetics11080537

**Published:** 2026-08-02

**Authors:** Jhon M. Pérez-Bohórquez, María C. Acero-Garzón, Sandra J. Gutiérrez-Prieto, Henry A. Méndez-Pinzón, Sandra J. Perdomo-Lara, Hernán Rodríguez-Hernández, María B. Solís-Valencia, Luis G. Sequeda-Castañeda

**Affiliations:** 1 Centre for Dental Research, Faculty of Dentistry, Pontificia Universidad Javeriana, Bogotá 110231, Colombia; jhonperez@javeriana.edu.co (J.M.P.-B.);; 2 Department of Physics, Faculty of Science, Pontificia Universidad Javeriana, Bogotá 110231, Colombia; 3 Unit of Basic Oral Investigations, Faculty of Dentistry, Universidad El Bosque, Bogotá 110121, Colombia; 4 Department of Chemistry, Faculty of Science, Pontificia Universidad Javeriana, Bogotá 110231, Colombia

**Keywords:** bone tissue engineering, 3D printing, human dental pulp stem cells (hDPSCs), finite element analysis (FEA), dental implants, hydroxyapatite/PLGA/Chitosan

## Abstract

Tooth loss and bone resorption of the alveolar cavity caused by dental caries and periodontal disease remain major clinical challenges that compromise oral function. Tissue engineering approaches based on biocompatible scaffolds have emerged as promising strategies for bone regeneration; however, the influence of fabrication methods on scaffold performance remains unclear. This study developed hydroxyapatite/poly (lactic-coglycolic acid)/chitosan scaffolds (HAp/PLGA/CS) using freeze-drying and 3D-printing techniques and evaluated their physicochemical, biological, and biomechanical properties. The morphology, porosity, elemental composition, and mechanical properties of the scaffold were characterized, while the biocompatibility and osteogenic potential were evaluated using human dental pulp stem cells (hDPSCs). Finite element analysis (FEA) using COMSOL Multiphysics^®^ Version 6.2. was performed to evaluate scaffold behavior under simulated dental implant loading conditions. The 3D-printed scaffolds exhibited significantly higher cell viability than the freeze-dried scaffolds, reaching approximately 85% in the 50% filling group compared with 50% in the freeze-dried group. Microstructural analysis revealed interconnected hierarchical porosity, including macro-, micro-, and submicrometer scale pores. Although the 50% infill scaffold showed the highest cell viability, the 70% infill scaffold demonstrated the most favorable osteogenic profile, with enhanced expression of RUNX2 and OSX. Both types exhibited degradation profiles compatible with early bone regeneration. FEA simulations indicated that further mechanical optimization is required to improve load transfer and reduce deformation at the implant–scaffold interface. Overall, HAp/PLGA/CS scaffolds showed potential as experimental bioactive platforms for bone tissue engineering, with 3D-printed scaffolds providing greater architectural control and favorable early osteogenic responses. However, the translational relevance of these findings remains preliminary and requires validation through long-term degradation studies, in vivo bone regeneration and osseointegration models, cyclic mechanical testing, and implant fixation experiments.

## 1. Introduction

Dental caries and periodontal disease are two of the most prevalent oral pathologies worldwide [1]. Both conditions can lead to partial or total tooth loss, significantly affecting masticatory function, facial esthetics, and dietary habits, while also inducing alveolar bone resorption that adversely affects the general health of the patient [1,2,3]. Although conventional prostheses are widely used to treat tooth loss, they often have long-term complications, including soft tissue hyperplasia, ulcerations, altered taste perception, and loss of proprioception [4]. Consequently, osseointegrated dental implants have revolutionized modern dentistry and markedly improved patient quality of life [5,6]. However, post-extraction bone remodeling frequently results in horizontal and vertical ridge defects, compromising implant esthetics and functionality while complicating placement. Restoring the dimensions of the alveolar ridge to ensure at least 1.5 to 2.0 mm bone thickness around the fixture is essential to achieve successful osseointegration [7].

Various techniques are used to regenerate bone tissue and meet these clinical requirements. Although autologous bone grafts remain the gold standard, their clinical utility is limited by limited donor site availability and the need for secondary surgical procedures [8]. Bone tissue engineering has emerged as a promising alternative, focusing on regeneration through the development of functional biological substitutes [8,9]. This discipline is based on a fundamental triad: growth factors, cells, and scaffolds. Scaffolds provide structural templates that support cell development and subsequent tissue formation. To be effective, they must be biocompatible, have interconnected porosity and adequate mechanical strength, and mimic the natural three-dimensional architecture of the extracellular matrix (ECM) [10].

In recent years, three-dimensional (3D) printing has enabled precise control over scaffold architecture, improving both mechanical performance and cytocompatibility. This technology also facilitates the incorporation of polymeric hydrogels, such as collagen, fibrin, and chitosan, which are essential to tailor the rheological, chemical, mechanical, and biological properties to better replicate the native ECM [11,12,13,14,15]. In parallel, calcium orthophosphate bioceramics, particularly hydroxyapatite (HAp) and beta-tricalcium phosphate (β-TCP), provide bioactive and osteoconductive mineral phases for bone regenerative scaffolds. However, their brittleness, low elasticity, and limited fatigue resistance restrict their use as standalone porous load-bearing materials [16,17,18]. Combining these ceramics with degradable polymers is therefore a rational strategy to improve processability, structural integrity, and architectural control while retaining mineral-mediated biological activity [16]. HAp/PLGA formulations have been investigated as cytocompatible systems for bone regeneration [16,19,20,21,22], while HAp/chitosan compounds can provide biodegradable and biomimetic matrices that support osteoblastic responses [16,23]. Recent evidence has extended this concept to ternary scaffolds for maxillofacial bone regeneration. Wang et al. (2024) [24] evaluated nHA/CS/PLGA scaffolds loaded with bone marrow stromal cells and stem cell exosomes in rabbit maxillofacial defects and reported improved bone repair. Because both cellular and exosomal components were incorporated, these findings support the feasibility of the ternary scaffold platform, but do not isolate the biological contribution of the scaffold itself [24].

Although freeze drying and 3D printing have already been investigated and compared in different bone scaffold systems, the comparison of these fabrication methods alone does not constitute the novelty of the present study. Previous investigations have generally examined different material compositions, conventional or synthetic calcium phosphate sources, nondental cell populations, or scaffold mechanics independently of implant-related loading conditions. Consequently, the integrated evaluation of a ternary HAp/PLGA/CS scaffold containing eggshell-derived HAp, fabricated by both freeze drying and 3D printing, biologically assessed using hDPSCs, and biomechanically analyzed under simulated dental implant loading remains insufficiently addressed. This research gap is particularly relevant for oral bone regeneration, where material origin, scaffold architecture, cellular response, mechanical properties, and load transfer at the implant–scaffold interface must be considered concurrently.

To address this research gap, HAp/PLGA/CS scaffolds were developed using two distinct fabrication techniques: freeze-drying [25] and 3D printing [26]. Their physical, structural, and mechanical properties were characterized with respect to porosity and elemental composition. Biocompatibility was assessed using human dental pulp stem cells (hDPSCs), which are readily accessible mesenchymal stromal cells with demonstrated osteogenic responsiveness to calcium phosphate and chitosan-containing matrices. Recent studies have shown that nanohydroxyapatite/carboxymethyl chitosan scaffolds support hDPSC viability and osteogenic differentiation and that dental pulp stem cell-seeded HAp scaffolds promote bone formation in preclinical maxillary defects [27,28,29,30].

Finally, to predict the ability of scaffolds to withstand the functional loads associated with oral implants, specifically the Brånemark™ oral implant system [31], and conduct a preliminary computational assessment of the mechanical feasibility of simultaneous scaffold and implant placement, finite element method (FEM) simulations were performed using COMSOL Multiphysics^®^ [32]. FEM is a computational approach that divides a complex three-dimensional structure into small elements and numerically solves the governing equations. In this study, the simulations incorporated scaffold geometry; material properties, including elastic modulus, density, and Poisson’s ratio; and boundary conditions representing fixed regions and load-bearing surfaces. The resulting stress and deformation maps allowed the identification of critical regions, elastic limits, and potential failure zones, providing information on the mechanical viability of the proposed scaffold–implant system. Recent computational approaches have extended finite element analysis from post hoc mechanical assessment to scaffold design optimization. Full-scale topology optimization can simultaneously adjust material distribution, apparent stiffness, and porous architecture to approximate the mechanical behavior of surrounding tissue while preserving the morphological features required for bone ingrowth [33,34]. This approach provides a relevant framework for future optimization of HAp/PLGA/CS scaffolds, in which the infill density, pore interconnectivity, mineral distribution, and degradation-dependent mechanical properties could be treated as coupled design variables. Consequently, the distinctive contribution of this study lies in the integration of HAp derived from sustainable eggshells, a ternary HAp/PLGA/CS composition, fabrication-dependent scaffold architecture, hDPSC-mediated biological evaluation, and implant-related finite element analysis within a single experimental framework.

## 2. Materials and Methods

The experimental design integrated the synthesis of HAp/PLGA/CS composites, scaffold fabrication through freeze-drying and 3D printing, and comprehensive structural characterization. In vitro biocompatibility was evaluated using human dental pulp stem cells (hDPSCs), while mechanical performance under functional loading conditions was evaluated through combined stiffness measurements (Young’s modulus), compressive strength, and microhardness, complemented by finite element analysis (FEA).

### 2.1. Development of HAp/PLGA/CS Shards

#### 2.1.1. Freeze-Drying Method

Hydroxyapatite (HAp) was obtained from avian eggshells through a combined thermal and chemical treatment according to the protocol reported by Sequeda et al. (2012) [35], which allows the conversion of calcium carbonate (CaCO_3_) into HAp. Poly (lactic-co-glycolic acid) (PLGA; 50:50 ratio, Mw 24,000-38,000; ref.: 719870) and high-molecular-weight chitosan (CS; Mw 310,000-35,000 Da; ref.: 419419) were purchased from Sigma-Aldrich (St. Louis, MO, USA)

Using these components, hydroxyapatite/poly (lactic-co-glycolic acid)/chitosan (HAp/PLGA/CS) composites were synthesized. The relative mass proportion of the three scaffold-forming components was established in a nominal HAp:PLGA:CS ratio of 1:4:8. In the freeze-drying formulation, the rounded concentrations relative to the total formulation were 1.07 wt% HAp, 4.34 wt% PLGA, and 8.69 wt% CS, corresponding to an approximate normalized ratio of 1:4.06:8.12. Cylindrical scaffolds (7 mm × 20 mm) were manufactured using the freeze-drying technique described by Wang [36] and Raman [25]. During this process, optimal proportions were established, and the resulting scaffolds were evaluated in terms of their physical and structural properties. Briefly, the suspension was cast into cylindrical molds and transferred directly to a static freezer at −70 °C for 48 h, followed by an additional 24 h at the same temperature. Because a programmable controlled-rate freezing system was not used, the cooling rate was not monitored and cannot be reported quantitatively. The frozen samples were subsequently lyophilized for 48 h at a chamber pressure of 0.470 mbar using a LABCONCO FreeZone 4.5 L benchtop freeze-dryer with a condenser temperature of −51 °C. The resulting freeze-dried cylindrical scaffolds were used for subsequent characterization.

#### 2.1.2. 3D-Printing Method

To fabricate HAp/PLGA/CS scaffolds by 3D printing, xanthan gum was incorporated into the previously optimized composite formulation (derived from freeze-dried scaffolds) to act as a rheological modifier, improving viscosity and printability. The nominal HAp:PLGA:CS mass ratio was maintained at 1:4:8 in the 3D-printing formulation. The corresponding rounded concentrations relative to the total printing formulation were 1.89 wt% HAp, 7.55 wt% PLGA, and 15.09 wt% CS, equivalent to an approximate normalized ratio of 1:3.99:7.98. Xanthan gum and water modified the absolute composition of the printing ink but did not alter the relative proportion between HAp, PLGA, and CS.

The scaffolds were printed using a BioX bioprinter (CELLINK, Gothenburg, Sweden) at an extrusion pressure of 20 kPa and a printing speed of 2.0 mm/s. A nozzle with an internal diameter of 0.580 mm was used. The nozzle and syringe temperatures were maintained at 36 °C, the print-bed temperature was maintained at 40 °C, and an initial stabilization period of 5 min was applied before printing. The programmed layer height was set at 0.300 mm, which corresponds to approximately 50% of the diameter of the nozzle. This setting ensured adequate interlayer adhesion and resolution while maintaining a consistent extrusion flow.

To investigate the effect of internal architecture, the printing parameters were adjusted to produce scaffolds with three infill densities (50%, 60%, and 70%), enabling controlled variation in porosity and filament distribution. Following fabrication, all scaffolds underwent a final freeze-drying step to remove the solvent phase, resulting in a stable and highly porous structure that mimics trabecular bone.

### 2.2. Physical and Structural Characterization of Both Types of Shackles

Following fabrication, the HAp/PLGA/CS scaffolds produced by both methods were evaluated in terms of their physical and structural properties, including porosity (pore size, distribution, density, and interconnectivity) as well as mechanical and chemical characteristics.

#### 2.2.1. Total Porosity

Total porosity was determined using the liquid displacement method [37]. This method is based on the relationship between the volume of fluid absorbed and the total volume of the scaffold, as defined by Equation (1):

(1) $$Porosity\left(\%\right)=\frac{\left(V_{2}-V_{3}\right)}{\left(V_{1}-V_{3}\right)}\times 100$$

where $V_{1}$ is the initial volume of ethanol (ACS, ISO, Reag. Ph Eur; Merck, Darmstadt, Germany), $V_{2}$ is the volume of ethanol with the scaffold immersed, and $V_{3}$ is the residual ethanol volume after scaffold removal.

#### 2.2.2. Pore Distribution and Interconnectivity

The composite porosity was further analyzed on morphological features using scanning electron microscopy (SEM). The samples were coated with a 15 nm gold layer using a Baltec SCD 050 system. Imaging was performed at an accelerating voltage of 10 kV under high-vacuum conditions and at multiple magnifications to evaluate different regions of the scaffold. Quantitative analysis of pore size distribution and interconnectivity was performed using the MATLAB software R2023a (MathWorks, Natick, MA, USA) [38]. SEM micrographs were converted to grayscale images to determine the diameter and depth of the pores, incorporating the pixel-to-micrometer conversion factor for accurate dimensional analysis. Subsequently, statistical analysis was conducted to evaluate pore density and interconnectivity.

#### 2.2.3. Mechanical and Chemical Characterization

The structural properties of the scaffolds, including hardness and elasticity, were initially estimated on the basis of the stoichiometric composition of CaO and HAp, followed by the analysis of the final tri-composite material. Elemental composition was determined by X-ray fluorescence (XRF) spectroscopy using a Shimadzu XRF-7000 instrument (Shimadzu, Kyoto, Japan).

The relationship between scaffold porosity, composition, and mechanical performance was evaluated by determining Young’s modulus, compressive strength, microhardness, and density. Cylindrical samples (7 mm diameter × 20 mm height) were tested in compression using a universal testing machine (MRC UTM-65A, MRC Laboratory Equipment, Holon, Israel). Three independently manufactured specimens per formulation or infill group (*n* = 3) were evaluated. Axial compression was applied at 0.5 N/s until structural failure or the onset of the densification plateau. The load and displacement were continuously recorded and converted into engineering stress and strain using the initial cross-sectional area and height of each specimen, respectively. Stress–strain curves were generated individually. Young’s modulus was determined exclusively by linear regression of the initial approximately linear region within the selected 5–30% relative strain interval. Compressive strength was defined as the maximum stress reached before failure or densification. Scaffold density was determined separately from the mass-to-volume ratio of each sample. The experimentally determined Young modulus and density values were subsequently incorporated as independent input parameters into the finite element analysis to evaluate the behavior of the scaffold under simulated implant-loading conditions.

Microhardness measurements were performed using a Vickers microhardness tester (Laizhou Lyric, Yantai, China) with a square-based pyramidal indenter applied at a penetration rate of 20 µm/s and a dwell time of 15 s. The Vickers hardness number (HV) was calculated using Equation (2):

(2) $$HV=\;\frac{F}{A_{s}}$$

where *F* is the load expressed in kilograms-force (kg-f), *As* is the surface area of the indentation (mm^2^), and *HV* is the Vickers microhardness number expressed in units of pressure.

#### 2.2.4. Energy-Dispersive X-Ray Spectroscopy (EDX) Analysis

Elemental analysis was also performed using energy-dispersive X-ray spectroscopy (EDX) coupled to a TESCAN LYRA3 field-emission scanning electron microscope (FE-SEM, TESCAN, Brno, Czech Republic). This technique is based on the emission of characteristic X-rays generated by the interaction between the incident electron beam and the sample. EDX spectra were collected from the same regions previously examined by SEM to ensure consistency between the morphological and compositional analyses.

### 2.3. Biocompatibility Evaluation of HAp/PLGA/CS Supports

#### 2.3.1. hDPSC Cell Culture

The biocompatibility of the scaffold was evaluated using commercially available primary human dental pulp stem cells (hDPSCs; Poietics™ Human Dental Pulp Stem Cells, catalog no. PT-5025; Lonza, Walkersville, MD, USA). According to the manufacturer, the cells were isolated from the dental pulp of adult human third molars obtained during tooth extraction. The hDPSCs were cultured in DPSC BulletKit™ medium (catalog no. PT-3005; Lonza, Basel, Switzerland) following the manufacturer’s instructions. A vial containing 5.0 × 10^5^ cells was evenly distributed between two 25 cm^2^ culture flasks, corresponding to 2.5 × 10^5^ cells per flask.

The culture medium consisted of 440 mL of base medium supplemented with 50 mL of DPSCGS, 10 mL of L-glutamine, 5 mL of ascorbic acid, and 0.5 mL of gentamicin/amphotericin. Cells were kept at 37 °C in a humidified atmosphere containing 5% CO_2_, and the medium was refreshed every 3 days.

Upon reaching approximately 80% confluence, cells were subcultured by trypsinization followed by centrifugation at 1800 rpm for 3 min. Cell concentration was determined using a Neubauer counting chamber according to Equation (3):

(3) $$Cell\;concentration=\;\left(\frac{Number\;of\;viable\;cells}{100}\right)\times dilution\;factor\times 10,000$$

Cells in passage 5 were subsequently seeded on scaffolds for biocompatibility evaluation.

#### 2.3.2. Biocompatibility Assessment of hDPSC-Seeded Sheets

The columns were sectioned into cylindrical specimens (4 mm in diameter and 2 mm in height) using a dermal biopsy punch and placed in 24-well plates. Samples were sterilized under ultraviolet (UV) light in a laminar flow hood for 15 min.

A total of 1.0 × 10^5^ cells per well were seeded onto the scaffolds in culture medium. Freeze-dried scaffolds were designated as L1 and L2, while 3D-printed scaffolds were categorized according to infill density (50%, 60%, and 70%).

Cell viability and adhesion, LDH release, osteogenic gene expression, and Alizarin Red S staining were evaluated after 7 days of culture. LIVE/DEAD and LDH analyses included freeze-dried scaffolds and 3D-printed scaffolds with 50%, 60%, and 70% infill, while RT-qPCR and Alizarin Red S analyses were performed using the experimental groups specified in the corresponding subsections.

Lyophilized bovine bone (HB; Fundación Cosme y Damián, Bogotá, Colombia) was used as an osteogenic reference material, and scaffold-free hDPSCs served as a control group for relative gene expression analysis [39].

#### 2.3.3. Cell Viability Analysis by Fluorescence Microscopy

After 7 days of culture, cell viability and adhesion were assessed using the LIVE/DEAD TM viability/cytotoxicity kit (Invitrogen TM, Waltham, MA, USA) following the manufacturer’s protocol. The samples were incubated for 10 min at 37 °C and subsequently observed using an Olympus IX71 fluorescence microscope.

The quantification of viable and nonviable cells was performed using ImageJ 1.12 software. All types of scaffolds (freeze-dried and 3D-printed with 50%, 60%, and 70% infill) were analyzed in triplicate.

In addition, cytotoxicity was evaluated using a lactate dehydrogenase (LDH) colorimetric assay (Roche). After 7 days, cell culture supernatants were collected, and absorbance was measured using a Tecan^®^ microplate reader. The LDH results were analyzed independently from the fluorescence imaging data.

#### 2.3.4. Osteogenic Gene Expression Analysis

After 7 days of culture, osteogenic gene expression was evaluated. Total RNA was extracted using the Quick-RNA kit (Zymo Research, Irvine, CA, USA), and the RNA concentration was measured with a NanoDrop 2000 spectrophotometer (Thermo Scientific, Waltham, MA, USA).

The experimental groups included freeze-dried scaffolds (L1 and L2) and 3D-printed scaffolds (60% and 70% infill). Lyophilized bovine bone (HB) was used as a reference material, and scaffold-free hDPSCs served as the control.

Gene expression was quantified by RT-qPCR using the Luna^®^ Universal One-Step RT-qPCR Kit (New England Biolabs, Ipswich, MA, USA). The GAPDH housekeeping gene was used as an internal reference, and relative gene expression was calculated using the $2^{-∆∆C_{t}}$ method.

The following osteogenic markers were analyzed: runt-related transcription factor 2 (RUNX2), osterix (OSX/SP7), alkaline phosphatase (ALP), collagen type I (COL1), and osteopontin (OP/SPP1). The primer sequences are listed in Table 1.

#### 2.3.5. In Vitro Mineralization Assay

In vitro bone mineralization was evaluated after 7 days of culture by Alizarin Red staining of calcium deposits. The columns were fixed with 10% formaldehyde and washed with PBS prior to application of the staining solution. After excess dye was removed through additional washing steps, representative images were captured. Subsequently, 10% acetic acid was added for 30 min to elute the calcium-bound dye. Finally, the supernatant was transferred to a new plate, and absorbance was measured at 490 nm using a Tecan^®^ spectrophotometer.

### 2.4. Finite Element Analysis (FEA) of Implant-Loaded HAp/PLGA/CS Scaffolds

#### 2.4.1. Mechanical Property Evaluation of HAp/PLGA/CS Scaffolds

Young’s modulus and density of the HAp/PLGA/CS scaffolds were used as separate input parameters for the finite element models. Young’s modulus was experimentally determined from the slope of the linear elastic region of the compressive stress–strain curve, following the procedure described in Section 2.2.3. The scaffold density was calculated independently as the ratio between the mass and external geometric volume. Thus, the mass-to-volume ratio was used exclusively to determine density and not used to calculate Young’s modulus [40,41].

#### 2.4.2. Finite Element Modeling and Simulation

Finite element simulations were performed using COMSOL Multiphysics^®^ Version 6.2. to analyze the mechanical response of scaffolds under loading conditions, both in the presence and absence of a dental implant. The implant was modeled as a titanium structure based on the Brånemark™ System, incorporating a porosity of 60%.

A solid mechanics study was conducted that assumed linear elastic behavior. The scaffold geometry was defined as a cylindrical model with a radius of 4 mm and a height ranging from 10 to 20 mm, consistent with the experimental methods.

The following material properties and boundary conditions were defined:

- Applied load (N);
- Elastic modulus (Pa) and Poisson ratio;
- Material density (kg/m^3^).

Boundary conditions were established by fixing the bottom surface of the scaffold, while a compressive load was applied perpendicular to the top surface along the negative *z*-axis.

Simulations allowed the evaluation of the stress distribution, deformation patterns, and overall mechanical performance, with particular emphasis on the implant–scaffold interface.

## 3. Results

### 3.1. Fabrication and Characterization of HAp/PLGA/CS Scaffolds

**Figure 1:**
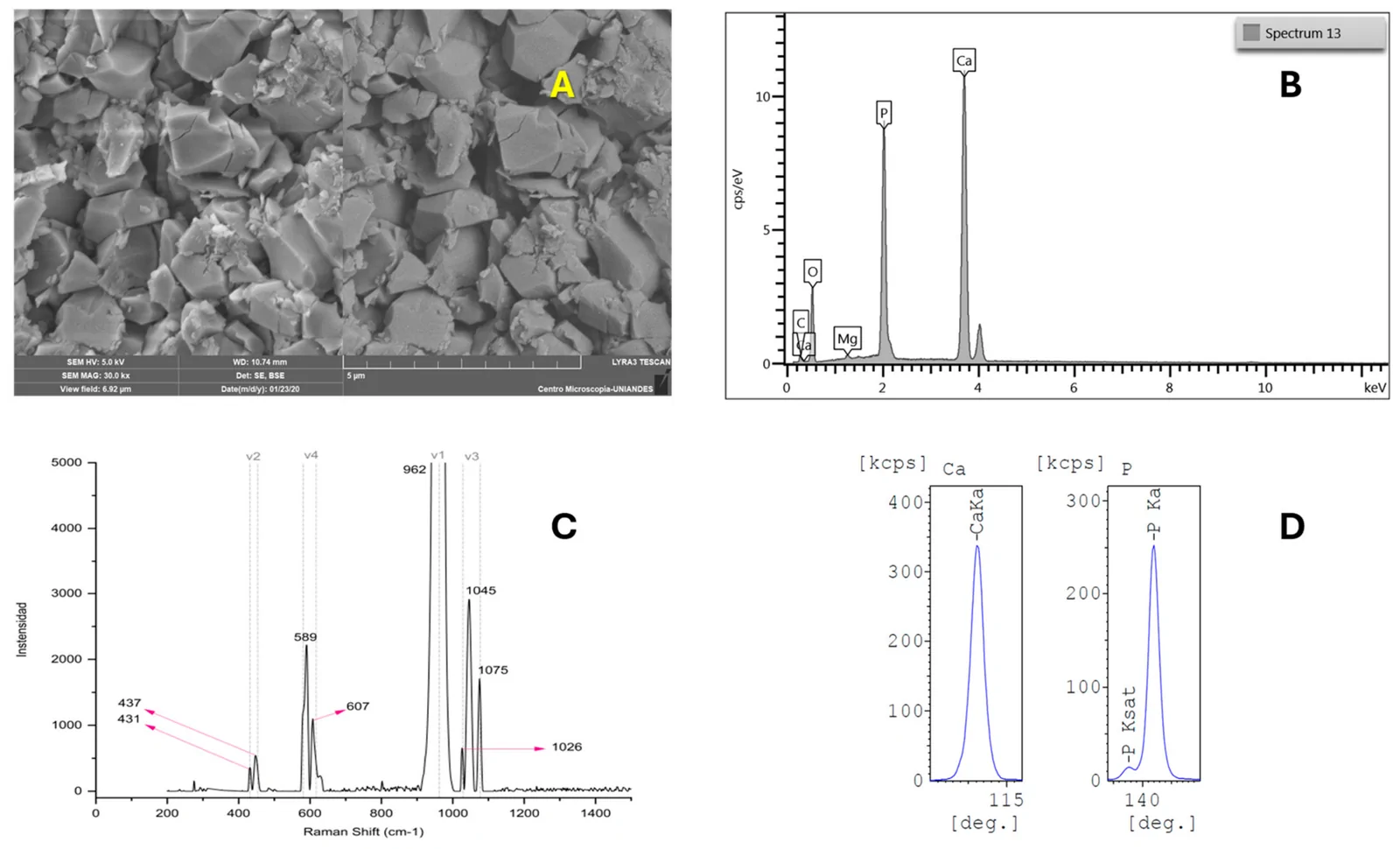
(**A**) Scanning electron microscopy (SEM, scale bar = 5 μm) images showing the polycrystalline surface morphology of sintered HAp, with an average grain size of approximately 1 μm. (**B**) Energy-dispersive X-ray spectroscopy (EDX) spectrum used for the elemental analysis of sintered HAp samples. (**C**) Raman spectrum of the material obtained after the sintering process. A prominent peak at 961 cm^−1^ is observed, which shows excellent agreement with the literature values of stoichiometric hydroxyapatite (~961 cm^−1^), corresponding to the symmetric stretching vibration mode (ν_1_) of the phosphate (PO_4_^3−^) group, which is characteristic of apatites [42]; red arrows highlight the low-intensity split modes of ν_2,_ ν_3_ and ν_4_. (**D**) X-ray fluorescence (XRF) spectrum of sintered HAp samples, showing calcium (Ca) and phosphorus (P) contents of 68.15% and 31.85%, respectively, yielding a Ca/P molar ratio of 1.65.

**Figure 2:**
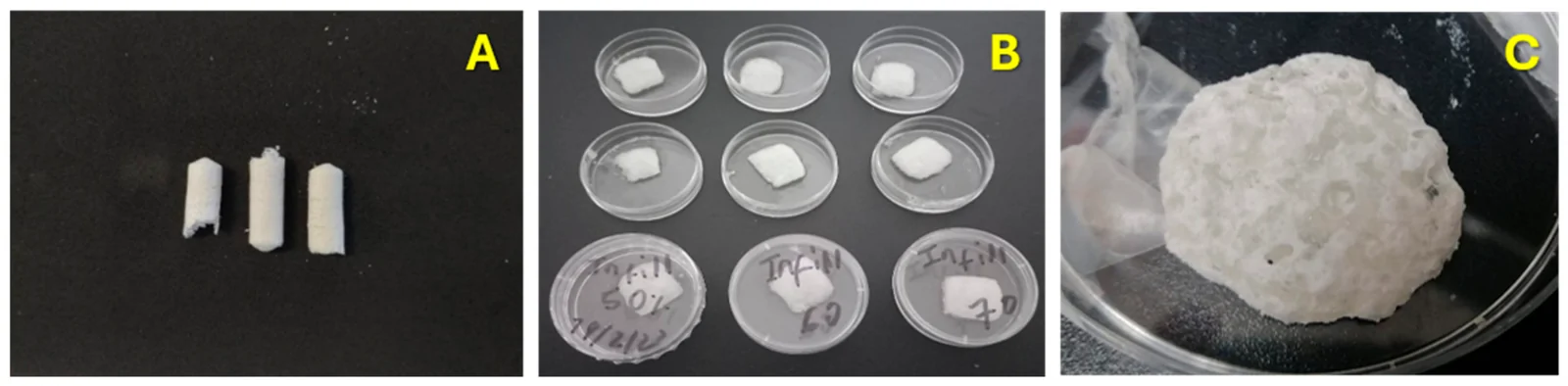
Macrostructure of the two types of scaffolds. (**A**) Macroscopic image of cylindrical HAp/PLGA/CS scaffolds fabricated using the freeze-drying method. (**B**) Initial macroscopic view of 3D-printed scaffolds with different infill densities (50%, 60%, and 70%). (**C**) Final macrostructure of 3D-printed scaffolds after lyophilization to remove the solvent phase, resulting in improved structural integrity and porosity, with morphological features that resemble trabecular bone.

The eggshell-derived material was characterized by scanning electron microscopy (SEM), energy-dispersive X-ray spectroscopy (EDX), Raman spectroscopy, and X-ray fluorescence (XRF), as shown in Figure 1A–D. SEM revealed a polycrystalline surface morphology, EDX and XRF confirmed the presence of calcium and phosphorus, and Raman spectroscopy showed the characteristic phosphate band of an apatitic calcium phosphate material. This biomaterial, which represents the main inorganic component of human bone, served as the primary precursor for the synthesis of the scaffolds developed in this study (Figure 2).

The final compositions used for the fabrication of HAp/PLGA/CS scaffolds by freeze drying and 3D printing are presented in Table 2 and Table 3, respectively. The percentage column corresponds to the amount of the added component (% *m*/*v*).

### 3.2. Physical and Structural Characterization of the Two Scaffold Types

#### 3.2.1. Morphology-Derived Porosity

**Figure 3:**
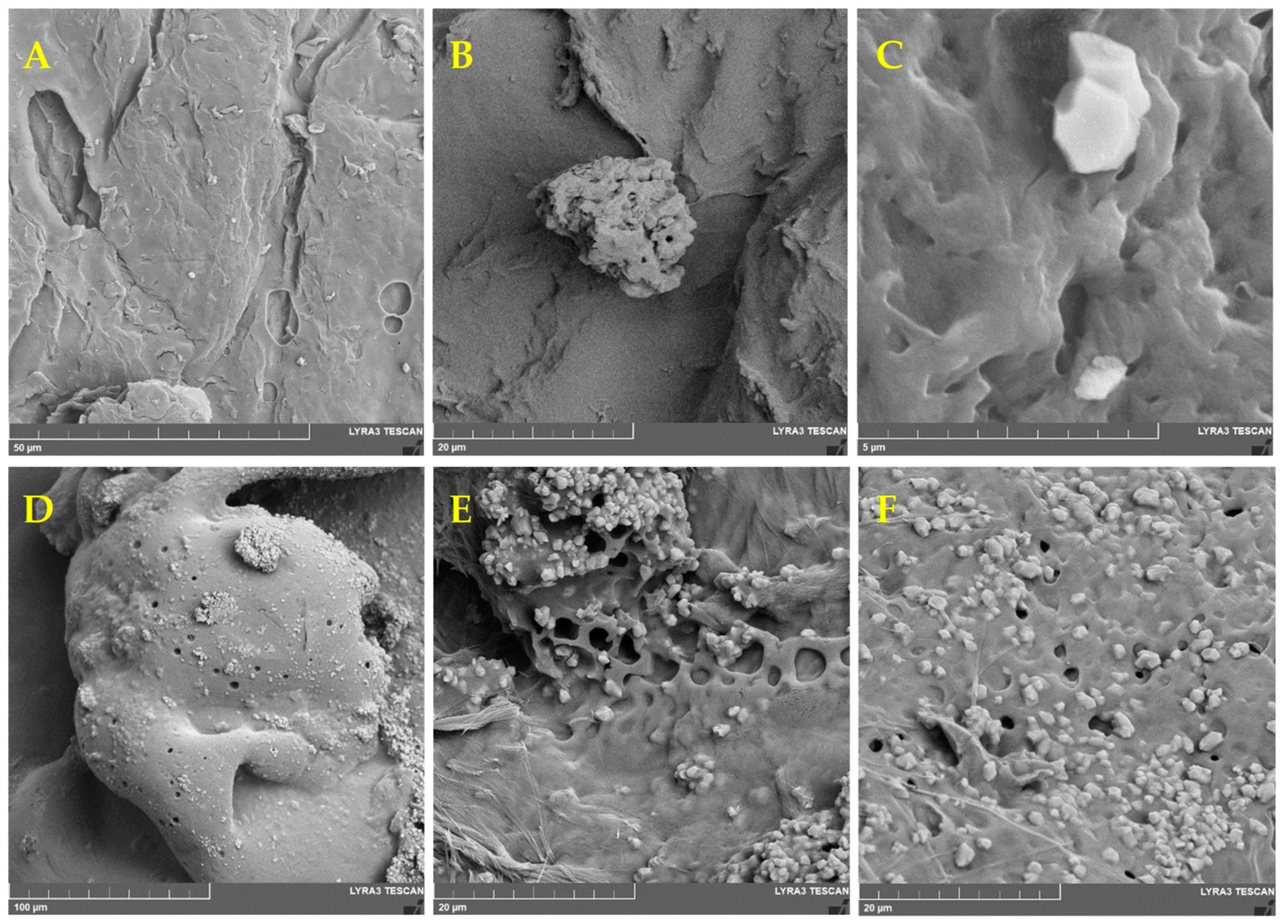
Scanning electron microscopy (SEM) micrographs of HAp/PLGA/CS scaffolds fabricated by the freeze-drying method (**A**–**C**) and 3D printing (**D**–**F**). (**A**) Large surface pores and smooth regions are observed, characteristics associated with the presence of PLGA within the scaffold matrix (scale bar = 50 μm). (**B**) Aggregated hydroxyapatite crystals that form cauliflower-like structures are evident (scale bar = 20 μm). (**C**) Well-defined crystals of hydroxyapatite (HAp) with characteristic hexahedral morphology are visible on the scaffold surface (scale bar = 5 μm). (**D**) SEM micrograph of a 3D-printed scaffold with 50% infill density, showing low surface roughness, smooth regions, and a heterogeneous distribution of small and large pores; in addition to microporosity, larger pores ranging from 180 to 500 μm are observed, indicating a hierarchical porous architecture (scale bar = 100 μm). (**E**) SEM micrograph of a 3D-printed scaffold with 60% infill density, exhibiting increased surface roughness, a higher density of relatively large pores, and fewer smooth regions compared to the 50% infill scaffold (scale bar = 20 μm). (**F**) SEM micrograph of a 3D-printed scaffold with 70% infill density, showing more uniformly distributed, smaller, and deeper pores, suggesting a more homogeneous porous architecture (scale bar = 20 μm).

SEM analysis revealed fabrication- and infill-dependent differences in scaffold morphology (Figure 3A–F). Freeze-dried scaffolds exhibited an irregular and heterogeneous pore network, with dense polymer-rich regions, discontinuous pores, and localized HAp aggregates. On the contrary, 3D-printed scaffolds showed progressively more controlled pore architectures as the infill density increased. The 50% infill scaffold combined larger pores, including voids of approximately 180–500 μm, with smaller surface pores; the 60% infill scaffold showed greater interconnectivity and surface roughness; and the 70% infill scaffold showed the most uniform distribution of smaller pores. Quantitative pore size, depth-map, and segmentation analyses are presented in Section 3.2.2 and Supplementary Figures S1 and S2 and Table S1.

#### 3.2.2. Pore Distribution and Interconnectivity

MATLAB-based image analysis of the pore distribution provided detailed information on the surface morphology of scaffolds fabricated by freeze-drying and 3D printing methods. Furthermore, depth-map reconstruction and binary image segmentation were used to quantitatively characterize pore architecture and spatial distribution within the scaffold structure (Supplementary Figure S1; Supplementary Table S1).

##### Freeze-Dried Scaffolds

To complement the qualitative SEM observations of the freeze-dried scaffolds (Figure 3A–C), a quantitative image-based porosity analysis was performed using MATLAB (Supplementary Figure S1; Supplementary Table S1). This analysis confirmed a predominantly microporous structure (dominant pore radius < 1 μm; overall range 0.2–3.5 μm) with sparse, discontinuous pore domains and limited interconnectivity, consistent with qualitative SEM interpretation. These results confirm that the freeze-drying process predominantly generates microporous structures with limited interconnectivity, supporting the qualitative interpretation from SEM analysis. The absence of a well-defined hierarchical pore network suggests that although these scaffolds may support initial cell attachment, their capacity for efficient mass transport, vascularization, and deep cell infiltration may be restricted compared to more architecturally controlled scaffolds fabricated via 3D printing. Full micrographs, depth maps, binary/pore space segmentations, and the corresponding pore size distribution histogram are provided in Supplementary Figures S1 and S2, with the complete quantitative summary in Supplementary Table S1.

##### 3D-Printed Scaffolds

Quantitative MATLAB-based image analysis of the 3D-printed scaffolds at 50%, 60%, and 70% infill densities (Supplementary Figures S1 and S2; Supplementary Table S1) con-firmed a progressive refinement of the pore architecture with increasing infill density: the 50% infill scaffold displayed a heterogeneous, hierarchical pore system that combined macropores (up to ~500 μm) and micropores (1–20 μm); the 60% infill scaffold showed a more homogeneous pore network (1–3.5 μm, dominant 1–2 μm) with increased intercon-nectivity; and the 70% infill scaffold exhibited the most uniform porosity dominated by submicron (0.2–1.5 μm, predominantly 0.2–0.7 μm). Full micrographs, depth maps, binary segmentations, and pore size distribution histograms for each infill density are provided in Supplementary Figures S1 and S2, with a complete quantitative summary in Supplementary Table S1.

The combined qualitative and quantitative analyses (Figure 3 and Supplementary Figures S1 and S2) demonstrate that the porosity of the scaffold is strongly dependent on the fabrication method and, in the case of 3D printing, infill density. The freeze-dried scaffolds (Figure 3A–C and Supplementary Figure S1) exhibited a highly heterogeneous and predominantly microporous structure, characterized by irregular pore geometry, limited interconnectivity, and a pore size distribution mainly below 3.5 μm. MATLAB-based segmentation confirmed that the pore domains were sparse and discontinuous, supporting SEM observations of dense regions interspersed with isolated pores. This type of porosity is typically associated with limited fluid transport and restricted cellular penetration beyond the scaffold surface.

On the contrary, the 3D-printed scaffolds (Figure 3D–F and Supplementary Figure S2) showed a progressive refinement of the pore architecture with increasing infill density. The 50% infill scaffolds (Supplementary Figure S2A) showed a hierarchical pore system that combined macropores (up to ~500 μm) and micropores (1–20 μm), as confirmed by SEM and depth mapping. This multiscale porosity is advantageous for bone tissue engineering, as macropores (>100 μm) facilitate vascularization and tissue ingrowth, while micropores improve protein adsorption and early cell attachment. However, the pore distribution at this infill level remained heterogeneous and less controlled.

At 60% infill (Supplementary Figure S2B), the pore network became more homogeneous, with a dominant pore size range of 1–3.5 μm and increased pore interconnectivity, as evidenced by the higher fraction of dark regions in binary segmentation images. This configuration suggests improved pathways for nutrient diffusion and cell migration, representing a balance between structural integrity and biological functionality.

The 70% infill scaffolds (Supplementary Figure S2C) exhibited the most uniform and refined porosity, with a narrow pore size distribution (0.2–1.5 μm) and a predominance of submicrometer pores. While this highly controlled microporous architecture may enhance the surface area for protein adsorption and osteogenic signaling, the reduction in macroporosity could limit deep cell infiltration and vascularization.

In general, these results highlight the trade-off between pore size, interconnectivity, and biological performance. Lower infill densities favor hierarchical porosity and improved mass transport, whereas higher infill densities promote structural uniformity and surface-driven biological interactions. The 3D printing approach, therefore, enables precise modulation of the scaffold architecture, allowing optimization of pore characteristics to balance mechanical stability with osteogenic potential.

### 3.3. Chemical Analysis of Scaffolds by Energy-Dispersive X-Ray Spectroscopy (EDX)

#### 3.3.1. Freeze-Dried Scaffolds

The elemental composition of the freeze-dried HAp/PLGA/CS scaffolds was evaluated using energy-dispersive X-ray spectroscopy (EDX), as shown in Supplementary Figure S3. The EDX spectrum (Supplementary Figure S3B) shows characteristic peaks corresponding to carbon (C), oxygen (O), phosphorus (P), and calcium (Ca), which are the main elements expected in the composite scaffold. Prominent signals associated with carbon and oxygen dominate the spectrum, reflecting the presence of the polymeric components (PLGA and chitosan), while distinct peaks corresponding to calcium and phosphorus confirm the incorporation of hydroxyapatite within the scaffold matrix. Additional minor peaks corresponding to gold (Au) are also observed, originating from the conductive coating applied during the SEM sample preparation.

Quantitative elemental analysis (Supplementary Figure S3A) indicates that carbon is the most abundant element, with a weight percentage of 60.34% (70.44 at.%), followed by oxygen at 29.11% (25.51 at.%). Calcium and phosphorus are present in lower proportions, with weight percentages of 7.05% (2.47 at.%) and 3.50% (1.59 at.%), respectively. The relative Ca and P content confirms the presence of a calcium phosphate phase within the scaffold, consistent with the expected composition of hydroxyapatite.

Overall, the EDX results demonstrate a composite structure dominated by the organic polymer matrix, with a homogeneous distribution of inorganic HAp components embedded within it.

For clarity and conciseness, the representative EDX spectrum and elemental-composition chart for the freeze-dried scaffold, corresponding to the quantitative results described above, have been moved to Supplementary Figure S3.

#### 3.3.2. 3D-Printed Scaffolds

The elemental composition of the 3D-printed HAp/PLGA/CS scaffolds (60% infill) was analyzed using energy-dispersive X-ray spectroscopy (EDX), as presented in supplementary Figure S4. The EDX spectrum (Supplementary Figure S4B) shows prominent peaks corresponding to oxygen (O), carbon (C), and calcium (Ca), which are consistent with the expected composition of the composite scaffold. Oxygen exhibits the highest intensity peak, followed by carbon, while a distinct calcium peak confirms the presence of the inorganic phase within the scaffold. As observed in the spectrum, minor peaks associated with gold (Au) are also present as a result of the conductive coating applied during sample preparation.

Quantitative analysis (Supplementary Figure S4A) showed that oxygen was the predominant detected element, accounting for 57.60 wt% (60.76 at.%), followed by carbon at 21.73 wt% (30.54 at.%) and calcium at 20.66 wt% (8.70 at.%). Phosphorus was not detected in the analyzed region. Because EDX provides localized surface information, this result should not be interpreted as demonstrating the absence of phosphorus throughout the scaffold. The nondetection may reflect heterogeneous mineral distribution, the limited area analyzed, or analytical constraints under the acquisition conditions; however, the available data do not allow these possibilities to be distinguished. Furthermore, the detection of calcium without a corresponding phosphorus signal does not independently confirm a calcium phosphate phase in that specific region. Replicated elemental mapping and complementary bulk and phase-specific analyses would be required to determine the spatial distribution and chemical form of the mineral component.

For clarity and conciseness, the representative EDX spectrum and elemental composition chart for the 3D-printed scaffold (60% infill), corresponding to the quantitative results described above, are included in Supplementary Figure S4.

A comparison of the EDX results obtained for scaffolds fabricated by freeze-drying (Supplementary Figure S3) and 3D printing (Supplementary Figure S4) reveals notable differences in elemental composition and relative phase distribution. The freeze-dried scaffolds exhibited a composition dominated by carbon (60.34 wt%) and oxygen (29.11 wt%), reflecting the high contribution of the polymeric matrix (PLGA and chitosan). On the contrary, calcium (7.05 wt%) and phosphorus (3.50 wt%) were present in relatively low proportions, consistent with limited exposure or dispersion of the hydroxyapatite phase within the scaffold surface analyzed by EDX.

Compared to 3D-printed scaffolds (Supplementary Figure S4), freeze-dried scaffolds showed a markedly different profile, characterized by a substantial increase in calcium content (20.66 wt%) and a higher proportion of oxygen (57.60 wt%), accompanied by a significant reduction in carbon content (21.73 wt%). These results suggest a greater relative contribution of the inorganic phase on the analyzed surface, likely due to the improved distribution or exposure of hydroxyapatite particles within the 3D-printed architecture.

From an atomic percentage perspective, the freeze-dried scaffold remained dominated by carbon-rich domains (70.44 at.%), while the 3D-printed scaffold displayed a more oxygen-rich composition (60.76 at.%) and a higher relative calcium content (8.70 at.% vs. 2.47 at.%).

Overall, these findings indicate that the fabrication method significantly influences the elemental composition of the surface, with freeze-drying producing a polymer-dominated surface and 3D printing promoting increased exposure of the mineral phase. This difference may have important implications for scaffold bioactivity, as higher calcium availability at the surface is typically associated with improved osteoconductivity and improved cellular response.

### 3.4. Biocompatibility Evaluation of HAp/PLGA/CS Freeze-Dried and 3D-Printed HAp/PLGA/CS Scaffolds

#### 3.4.1. Morphological and Immunophenotypic Characterization of hDPSCs

**Figure 4:**
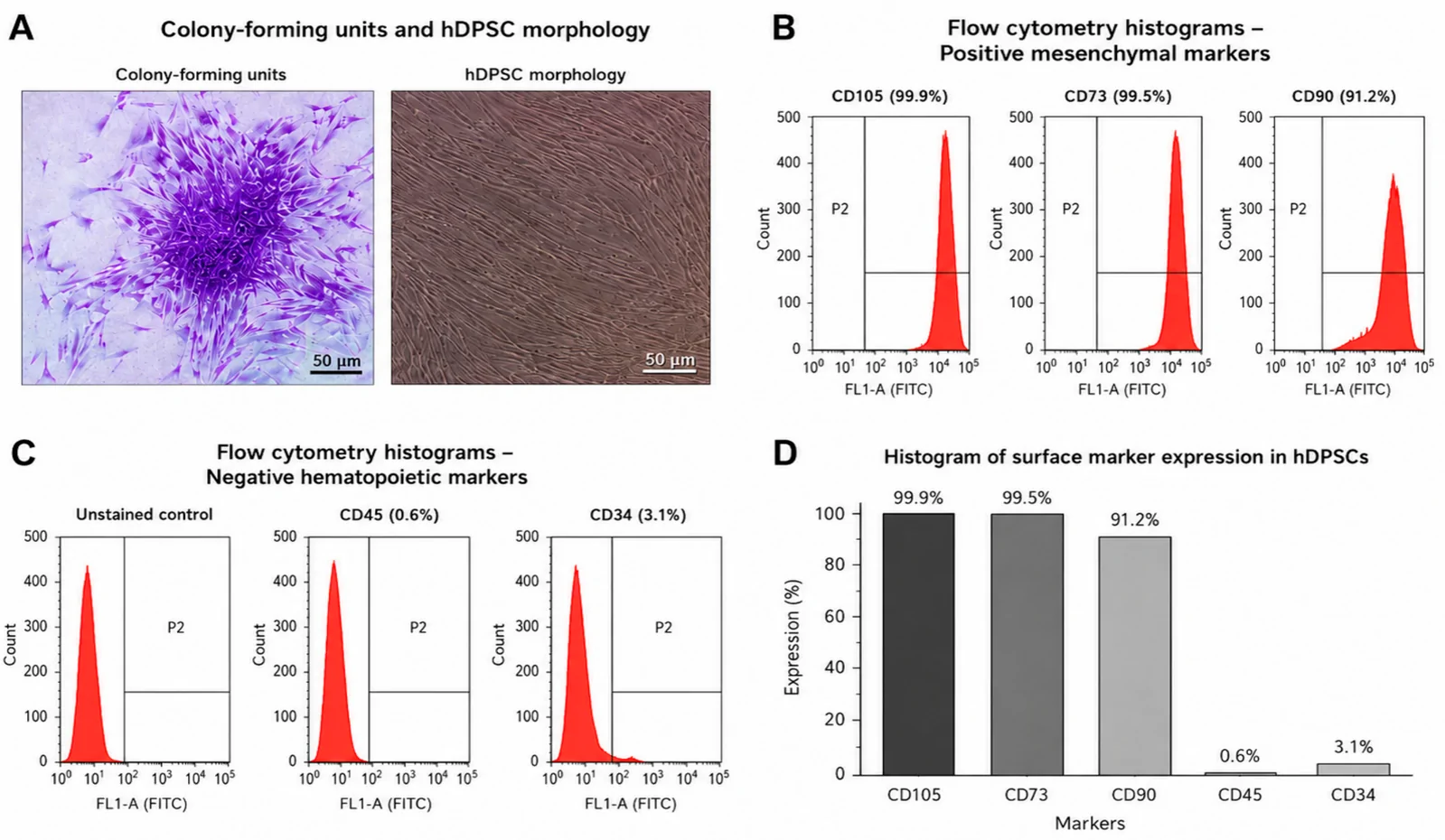
Morphological and immunophenotypic characterization of human dental pulp stem cells (hDPSCs). (**A**) Representative micrograph showing colony formation cells (CFCs) and fibroblast-like morphology of hDPSCs in culture (scale bar = 50 μm). (**B**) Flow cytometry histograms illustrating the expression of positive mesenchymal markers CD105, CD73, and CD90. (**C**) Flow cytometry histograms showing the unstained control and low expression of negative hematopoietic markers CD45 and CD34. (**D**) Quantitative summary of surface marker expression in hDPSCs.

On day 7 of culture, human dental pulp stem cells (hDPSC) were observed adhering to the surface of the culture flasks, exhibiting an elongated fibroblast-like morphology, monolayer organization, and growth characteristics consistent with mesenchymal stem/stromal cells (MSCs). Furthermore, the structures of colony-forming cells (CFCs) were identified as dense cellular clusters composed of spindle-shaped cells radially organized from a central core, confirming the clonogenic potential of the evaluated cell population (Figure 4A).

Immunophenotypic characterization by flow cytometry demonstrated high expression of markers associated with mesenchymal stromal cells. Histograms revealed a clear shift of the cell population towards the positive region, with expression levels of 99.9% for CD105, 99.5% for CD73, and 91.2% for CD90 (Figure 4B). These findings indicate that the hDPSC population maintained a predominantly mesenchymal phenotype under the culture conditions used.

On the contrary, the hematopoietic markers CD45 and CD34 exhibited low expression levels of 0.6% and 3.1%, respectively (Figure 4C). The low expression of these markers, together with the high positivity for CD105, CD73, and CD90, confirms that the cell population displays an immunophenotype consistent with mesenchymal stem/stromal cells (Figure 4D).

In general, morphological and immunophenotypic analyses confirm that hDPSCs used in the biocompatibility assays of HAp/PLGA/CS scaffolds retain the defining characteristics of mesenchymal stem/stromal cells.

#### 3.4.2. Evaluation of Cell Viability and Adhesion in HAp/PLGA/CS Scaffolds by Fluorescence Optical Microscopy

The live/dead assay was performed after 7 days of culture to evaluate hDPSC survival and adhesion after sustained interaction with the HAp/PLGA/CS scaffolds. This time point was selected to assess cell response in the same culture period used for osteogenic gene expression analysis.

**Figure 5:**
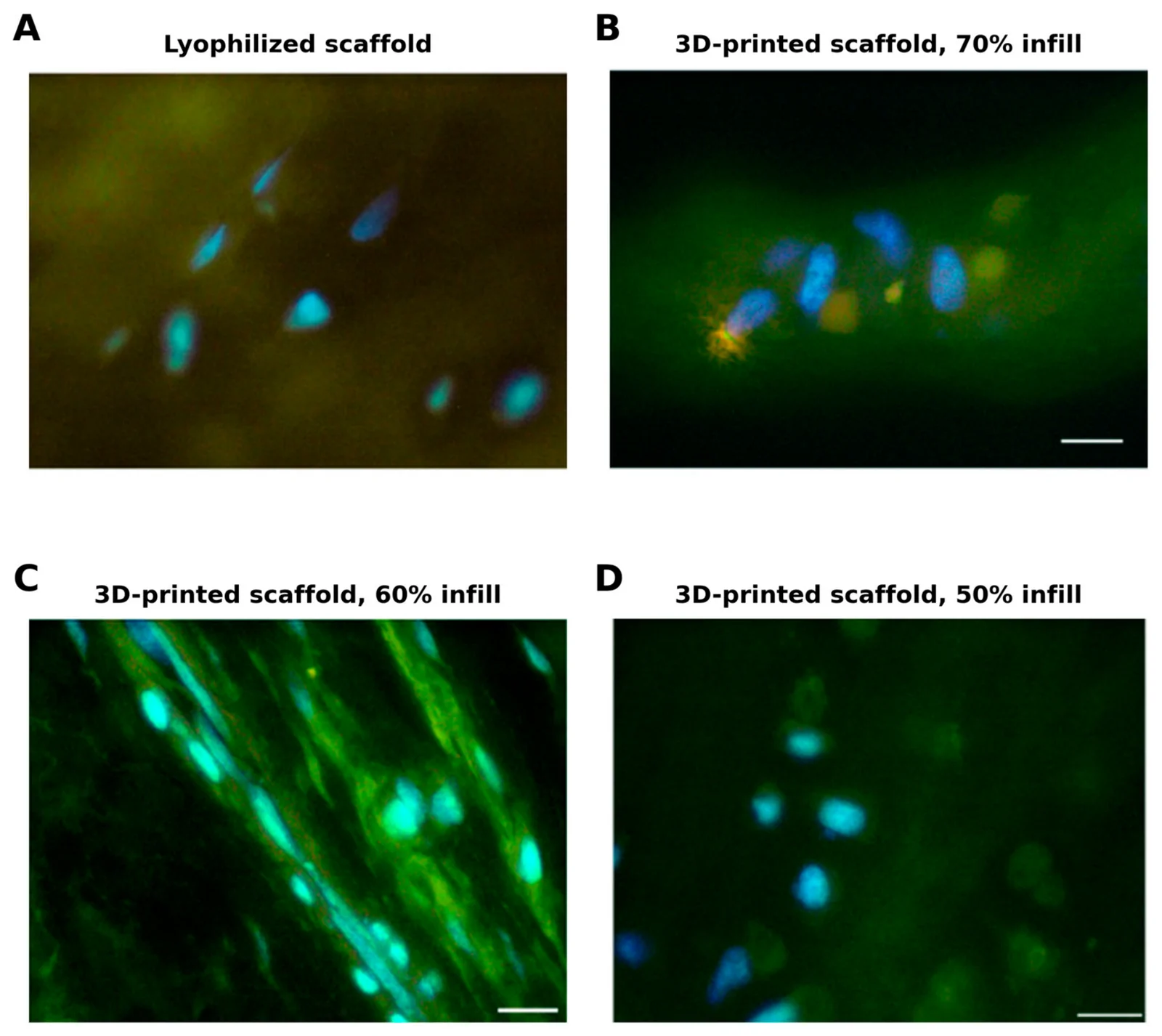
Fluorescence microscopy analysis of cell viability on HAp/PLGA/CS scaffolds fabricated by freeze-drying and 3D printing. (**A**) Freeze-dried scaffold. (**B**) 3D-printed scaffold with 70% infill density. (**C**) 3D-printed scaffold with 60% infill density. (**D**) 3D-printed scaffold with 50% infill density. Scale bars = 50 µm.

After 7 days of culture, the hDPSCs seeded in HAp/PLGA/CS scaffolds were evaluated using live/dead staining and quantitative viability analysis. Fluorescence imaging demonstrated the presence of viable cells adhered to freeze-dried and 3D-printed scaffolds with nominal infill densities of 50%, 60%, and 70% (Figure 5A–D).

In the freeze-dried scaffolds, the fluorescent cells were sparsely distributed on the surface, indicating a lower apparent cell density compared to the 3D-printed scaffolds (Figure 5A). In contrast, the 3D-printed groups exhibited a higher number of adherent viable cells, particularly in 50% and 60% infill scaffolds, where the cells were more evenly distributed and closely associated with the scaffold matrix (Figure 5C,D). In the 70% infill group, viable cells were also observed; however, their distribution appeared to be more localized within the analyzed field (Figure 5B).

Quantitative analysis revealed significant differences in cell viability between groups. The 3D-printed scaffolds exhibited the highest viability, reaching approximately 85% in the 50% infill group, followed by the 60% and 70% infill groups. In contrast, the freeze-dried scaffolds showed the lowest viability values, close to 50% (Supplementary Figure S5). Statistical analysis confirmed that cell viability in the freeze-dried group was significantly lower than in all 3D-printed scaffold groups (*p* < 0.05).

#### 3.4.3. Analysis of Bone-Related Gene Expression: RUNX2, Osterix (OSX), ALP, COL1, and Osteopontin (OPN)

**Figure 6:**
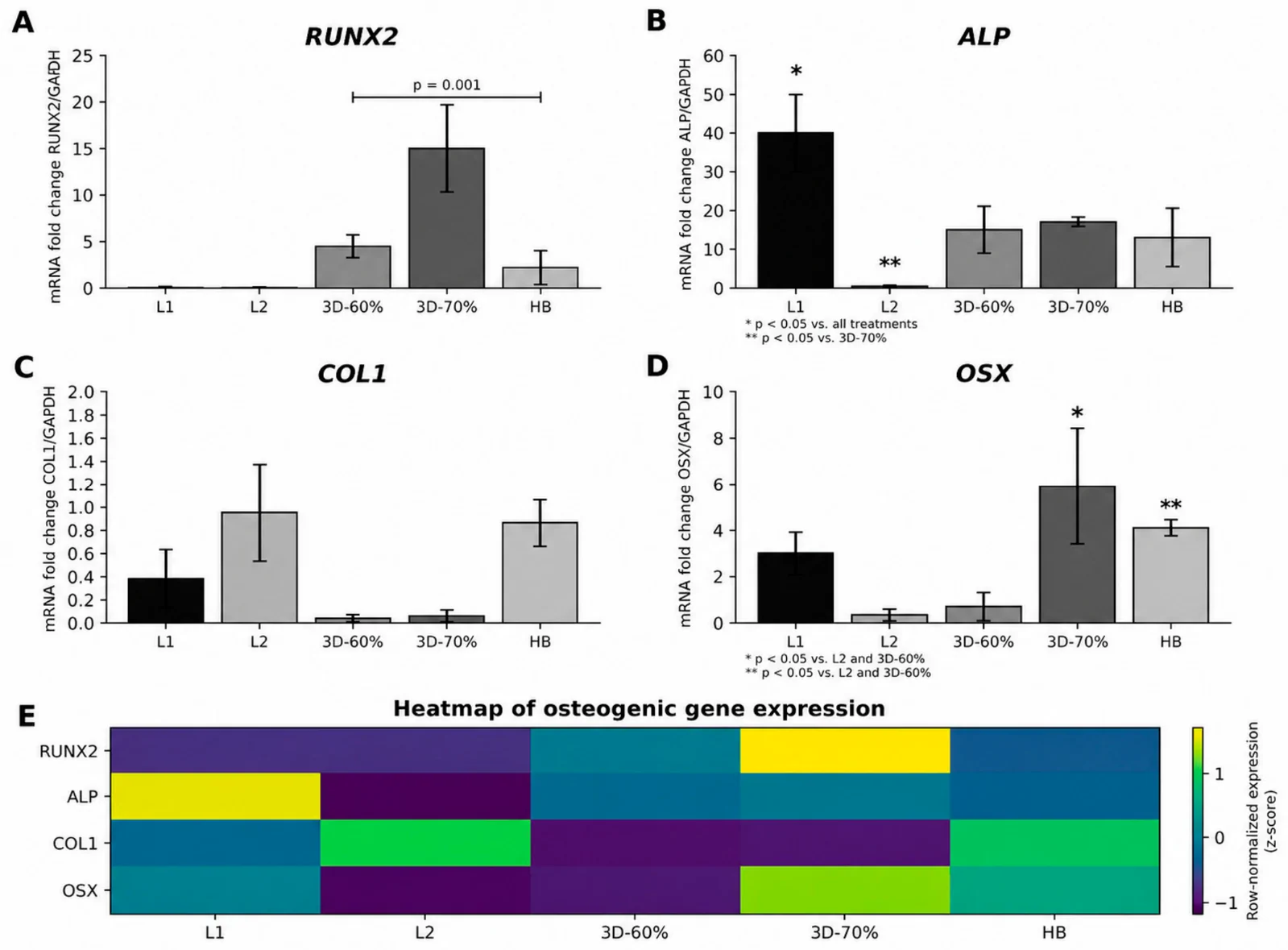
Expression of osteogenic differentiation-related genes in hDPSCs cultured on HAp/PLGA/CS scaffolds. Relative gene expression was quantified by RT-qPCR after 7 days of culture on freeze-dried and 3D-printed scaffolds. (**A**) Relative expression of RUNX2, an early marker associated with osteogenic commitment. (**B**) Relative expression of alkaline phosphatase (ALP), a marker related to early osteoblastic activity and matrix maturation. (**C**) Relative expression of type I (COL1), associated with the formation of extracellular matrix. (**D**) Relative expression of osterix (OSX), a transcription factor involved in osteoblastic differentiation. (**E**) Heatmap summarizing osteogenic gene expression between experimental groups, including freeze-dried scaffolds (L1 and L2), 3D-printed scaffolds with 60% and 70% infill densities, and freeze-dried bovine bone (BB) as a reference material. Bar graph data are presented as mean ± standard deviation. The heatmap shows gene-wise normalized expression to facilitate comparison among groups. Statistical significance was defined as *p* < 0.05, as indicated in each panel.

The expression of osteogenic genes in hDPSCs cultured for 7 days on HAp/PLGA/CS scaffolds exhibited different profiles between the experimental groups (Figure 6). RUNX2 expression was predominantly up-regulated in the 3D-printed scaffold with a 70% infill density (Figure 6A). On the contrary, the expression of alkaline phosphatase (ALP) was highest in the freeze-dried scaffold (L1), followed by the 3D-printed scaffolds with 60% and 70% infill densities, as well as the bovine bone control (Figure 6B).

Collagen type I (COL1) expression was higher on the L2 scaffold and in bovine bone, while lower expression levels were observed in the 3D-printed scaffolds (Figure 6C). Similarly, Osterix (OSX) expression was highest in 70% infill scaffold, followed by bovine bone and L1 scaffold (Figure 6D).

Heatmap analysis summarized the overall gene expression patterns, indicating that each scaffold formulation induced a distinct osteogenic response (Figure 6E). In general, the 3D-printed scaffold with 70% infill density exhibited the most favorable profile for early osteogenic markers, particularly RUNX2 and OSX, suggesting enhanced osteogenic differentiation potential under these conditions.

#### 3.4.4. Evaluation of Mineralized Nodule Formation by Alizarin Red S Staining

**Figure 7:**
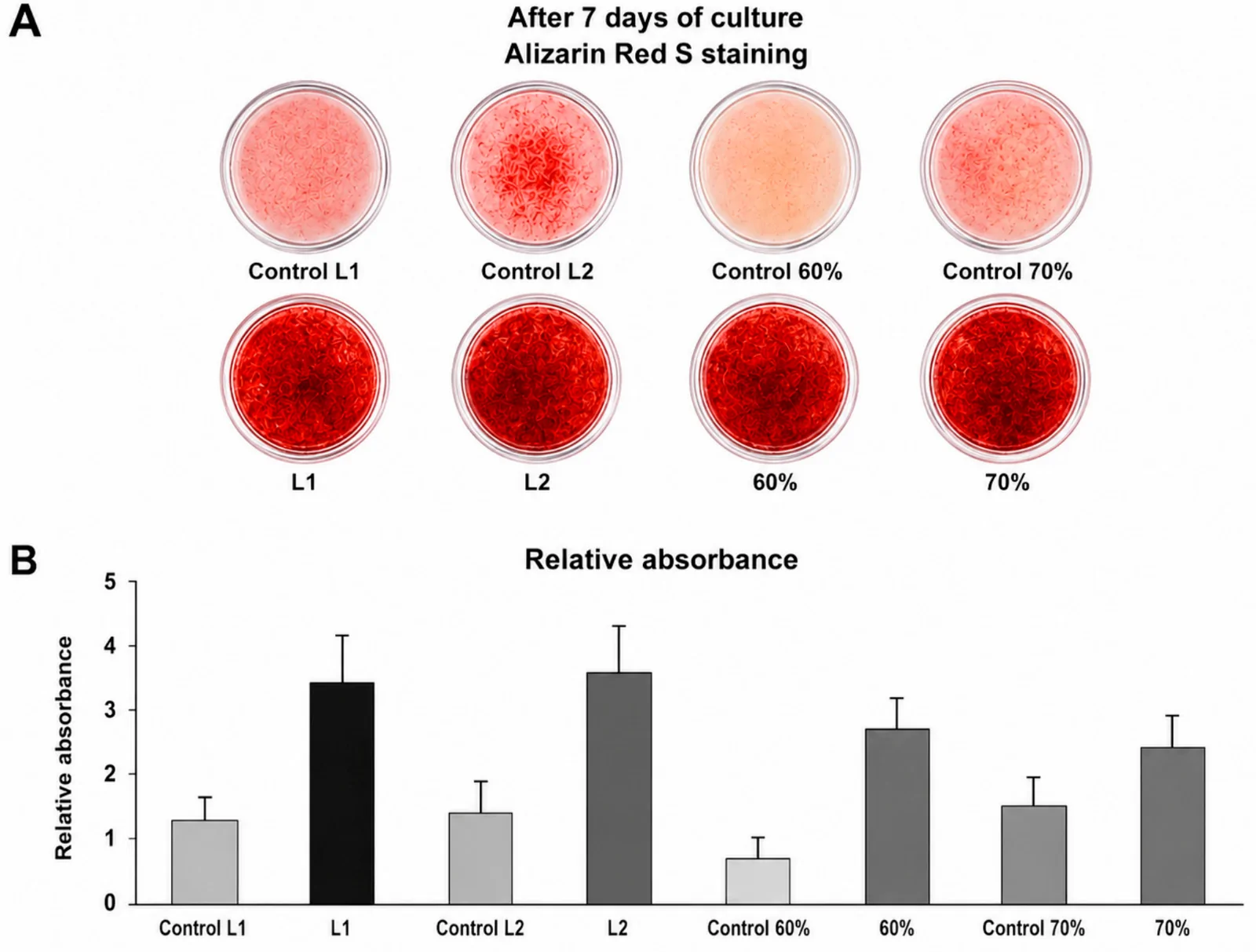
Evaluation of mineralized deposits in HAp/PLGA/CS scaffolds by Alizarin Red S staining. (**A**) Representative images of Alizarin Red S staining for the different experimental groups and corresponding controls, including freeze-dried scaffolds (L1 and L2) and 3D-printed scaffolds with 60% and 70% infill densities, together with their respective acellular controls. (**B**) Quantitative analysis of Alizarin Red S staining, expressed as relative absorbance of each group. Cell-seeded scaffolds exhibited higher absorbance values compared with those of their corresponding acellular controls. Data are presented as mean ± standard deviation.

Alizarin Red S staining produced positive signals in both acellular controls and scaffolds cultured with hDPSCs, as expected, as a result of the presence of hydroxyapatite within the scaffold composition. However, the experimental groups with cell membranes exhibited more intense staining and higher relative absorbance values compared to their corresponding acellular controls (Figure 7A,B).

### 3.5. Finite Element Analysis of the Mechanical Properties of HAp/PLGA/CS Scaffolds with and Without Implant Loading

The results of the mechanical simulation obtained using COMSOL Multiphysics reveal a significant variability in the stiffness of the unloaded scaffolds, with values ranging from 4 to 40 MPa. The highest stiffness value corresponded to a scaffold fabricated by the freeze-drying method (40 MPa), suggesting greater resistance to deformation associated with its specific internal architecture. In general, the scaffolds produced by freeze-drying exhibited higher stiffness values compared to those manufactured by 3D printing, although some overlap was observed in the intermediate range. On the contrary, 3D-printed scaffolds showed moderate stiffness values between 6 and 19 MPa, consistent with more porous and geometrically controlled structures (see Table 4).

Table 5 shows the data used as the basis for predicting the load on these implants and determining whether they meet the mechanical properties required to withstand this load.

Of the six scaffolds produced using the freeze-drying method, five were selected, corresponding to models 1 through 5. Of the four scaffolds produced using 3D printing, two were selected, corresponding to models 6 and 7. Data on the implants (Brånemark™ system oral implants) and bone were taken from the literature, using previously reported values for mechanical properties such as elastic modulus and density [40,43].

Among the 3D-printed scaffolds, the two models evaluated by FEA (models 6 and 7, Table 4) showed stiffness values of 6 and 19 MPa, respectively, directly reflecting the infill-dependent porosity trend described in Section 3D—Printed Scaffolds: the higher-stiffness model is consistent with a higher infill density and correspondingly reduced pore size, and porosity, whereas the lower-stiffness model corresponds to a lower infill density and a more open pore network. This indicates that increasing the density of the infill measurably increases scaffold stiffness, in agreement with the reduced macroporosity and more homogeneous sub-micrometer-dominated pore structure observed at higher infill (Section 3D—Printed Scaffolds).

**Figure 8:**
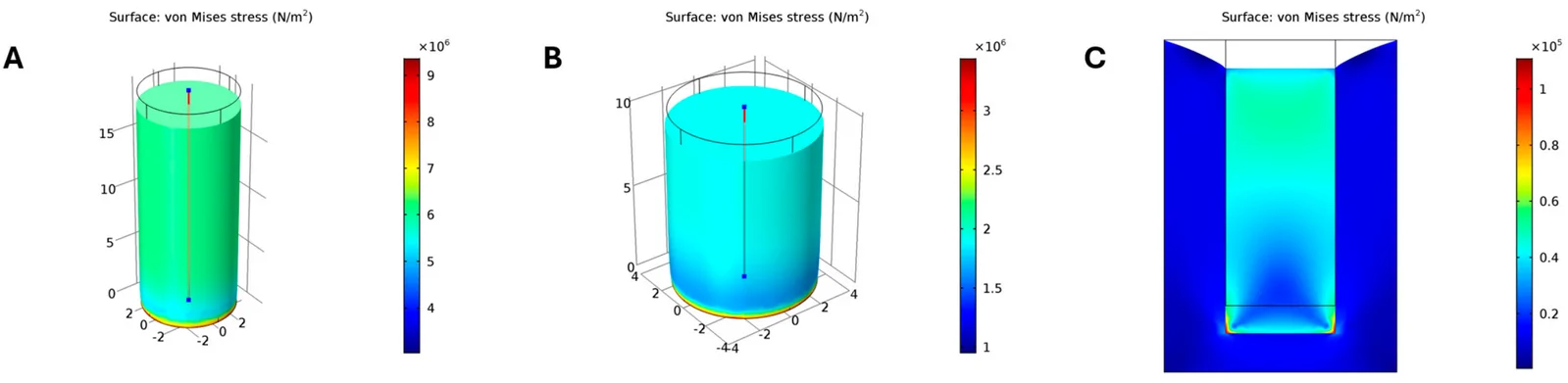
Images from the MEF simulation of the HAp/CS/PLGA scaffolds. The models are shown on a color scale that corresponds to the stress distribution along the cylinder’s axis, where red indicates the region of maximum deformation, which is also observed in the z-direction. The implant used is a titanium implant with 60% porosity (Branemark System). (**A**) Scaffold made using the freeze-drying method (model 4). (**B**) Scaffold manufactured using the 3D printing method (model 7). (**C**) Scaffold model 7 with a titanium implant.

Figure 8A,B show the results of the simulation of the von Mises stress distribution along the symmetry axis when a static compressive load (occlusal) is applied. Models 4 and 7 were selected, as they have the highest modulus of elasticity values for scaffolds manufactured by the freeze-drying method and 3D printing, respectively. In both cases, it can be observed that in most of the cylinders (green zone for model 4 and magenta zone for model 7), the stress stabilizes uniformly. The highest stress concentration occurs at the base of the cylinder (from z = 0 mm to approximately z = 3 mm). The material exhibits elastic behavior according to Hooke’s law. The highest stress values occur in the red region, exceeding 9 MPa for model 4 and approximately 3.5 MPa for model 7. This stress concentration could lead to a fracture or collapse precisely at the base, but not in the body of the cylinder.

Figure 8C shows a simplified two-dimensional simulation of a model 7 scaffold with a smooth titanium implant that has 60% porosity (Brånemark System). A force is applied to the implant in the z direction and, in turn, exerts a force on the scaffold. Therefore, a phenomenon of implant penetration into the scaffold is observed. The modulus of elasticity of the implant is 500 times greater than that of the scaffold. Due to the large difference in moduli, no “load” transfer from the implant to the scaffold is observed; this can be seen from the uniform blue color in the matrix. This phenomenon is known as “stress shielding”. Stress concentration occurs at the base of the implant and particularly at its edges; this phenomenon can lead to plastic deformation and, probably, scaffold fracture. On the other hand, at the surface of the matrix (z = 10 mm), distortion occurs, which means that the walls adjacent to the implant pull the scaffold downward as shear stress, deforming its geometry. The smooth metal implant transmits the load primarily through the lower edges of the base, resulting in potential local damage due to this stress concentration.

To achieve better load transfer from the implant to the scaffold, the modulus of elasticity must be increased to values close to those of the implant. Furthermore, the implant should not have smooth walls but rather a grooved surface, such as a screw. This ensures better load transfer and prevents localized stress concentration.

## 4. Discussion

The treatment of bone defects remains a significant clinical challenge. Despite advances in tissue engineering, no definitive solution has yet been achieved [44]. In this context, research has increasingly focused on the development of biocomposite scaffolds capable of promoting cell proliferation, differentiation, and subsequent bone tissue formation [45,46,47,48]. Among the most promising strategies are the freeze-drying method and 3D printing, which stand out for their ability to produce structures with adapted physical, mechanical, and morphological properties suitable for bone regeneration [27,45,46,47,48].

In the present study, HAp/PLGA/CS scaffolds were fabricated using freeze-drying and 3D printing techniques. Their physicochemical and mechanical properties were characterized, and their biocompatibility was evaluated using hDPSCs. The polymeric components were selected to address the intrinsic mechanical limitations of HAp. Although HAp is biocompatible, bioactive, and osteoconductive, calcium orthophosphate ceramics used alone exhibit brittleness, low elasticity, poor tensile strength, and low fatigue resistance; these limitations become more critical in highly porous scaffolds [16,46]. Incorporation of CS and PLGA provides a degradable polymeric matrix intended to improve processability and structural cohesion and allows the development of a composite architecture that more closely approximates the organic–inorganic organization of native bone [17,18,19,20,21,45,46,47,48]. Furthermore, finite element analysis (FEA) was used to predict scaffold behavior under implant-related loading conditions, with the objective of evaluating its potential to reduce oral rehabilitation times through the future implementation of simultaneous scaffold–implant placement [11,19,40].

The principal contribution of this study is therefore not the comparison between freeze-drying and 3D printing in isolation. Rather, it is the integration of eggshell-derived HAp within a ternary HAp/PLGA/CS scaffold, the evaluation of fabrication-dependent architecture and mechanical properties, the assessment of cytocompatibility and early osteogenic responses using hDPSCs, and the simulation of load transfer within a dental implant–scaffold system. This combined approach makes it possible to examine the relationships among sustainable material sourcing, scaffold microarchitecture, cellular behavior, and biomechanical compatibility, which are frequently studied separately in previous investigations.

A distinctive aspect of this study was the use of HAp obtained from eggshells, an abundant agro-industrial byproduct, through wet-chemical synthesis [19,35]. XRF analysis yielded a Ca/P molar ratio of 1.65. This value is close to the theoretical ratio of 1.67 for stoichiometric HAp, Ca_10_(PO_4_)_6_(OH)_2_ [49]. However, it also falls within the reported range for calcium-deficient hydroxyapatite (1.50–1.67); consequently, the Ca/P ratio alone cannot establish exact HAp stoichiometry or exclude the presence of a calcium-deficient apatite phase [49]. The Raman band at 961 cm^−1^, together with the Ca and P signals detected by XRF and EDX, supports the formation of an apatitic calcium phosphate material (Figure 1A–D). Because XRD data are not reported in the present study, these findings should be interpreted as supporting HAp-like apatite formation rather than providing definitive crystallographic phase identification. However, the results indicate that eggshell-derived calcium can be converted into an apatitic material with a Ca/P ratio comparable to those reported for eggshell-derived HAp [50,51]. Recent investigations further demonstrate that the biological and mechanical performance of eggshell-derived HAp is strongly dependent on composition and processing. Titanium-doped eggshell-derived HAp has shown biocompatibility and new bone formation in an animal model, while systematic thermal studies have demonstrated the temperature-dependent transformation of HAp into β-TCP and α-TCP, accompanied by substantial changes in density, hardness, compressive strength, and fracture toughness [52,53]. These findings reinforce the importance of reporting the thermal history and performing phase-specific structural characterization when comparing eggshell-derived HAp materials.

The use of eggshell-derived HAp in the present study was motivated by sustainable sourcing and agro-industrial waste rather than an expectation of superior performance relative to commercial Hap [54]. Because a commercial HAp control was not included, the present results do not establish equivalence or superiority in terms of phase composition, mechanical behavior, degradation, cytocompatibility, or osteogenic performance. Therefore, direct comparative studies using materials processed and evaluated under identical conditions will be required to determine whether eggshell-derived HAp provides functional advantages beyond its sustainability-related benefits [54].

During scaffold fabrication, HAp tended to form aggregates within the polymeric matrix, resulting in a heterogeneous mineral distribution. Particle agglomeration and inadequate adhesion at the calcium orthophosphate–polymer interfaces are recognized limitations of these composites because they can reduce phase homogeneity and compromise interfacial load transfer and mechanical performance [16,55]. In the present study, the relative content of HAp was reduced, and the CS and PLGA fractions increased (Table 2 and Table 3) as a formulation adjustment intended to improve material homogeneity. However, the dispersion of HAp and interfacial adhesion were not independently quantified, and the effect of composition cannot be separated from that of the fabrication method. Therefore, the improvement should not be described as a demonstrated causal consequence of changing the proportions of components. Future studies should quantify the particle size distribution, mineral dispersion, and interfacial adhesion and assess compatibilization or surface functionalization strategies.

The resulting scaffolds exhibited distinct porosity characteristics depending on the fabrication method. Quantitative image analysis (Supplementary Figures S1 and S2, Supplementary Table S1) revealed that the freeze-dried scaffolds were predominantly microporous, with pore sizes mainly below 3.5 μm, limited interconnectivity, and a heterogeneous spatial distribution. On the contrary, 3D-printed scaffolds showed a hierarchical and tunable pore architecture, with the pore size and distribution strongly influenced by the infill density. In particular, 50% of the infill scaffolds exhibited a multiscale pore system, including macropores (up to ~500 μm) alongside micro- and submicrometer pores, while increasing infill density (60% and 70%) resulted in a more homogeneous and refined microporous network (Figure 3, Supplementary Figures S1 and S2, Supplementary Table S1).

These observations are consistent with the known influence of fabrication techniques on scaffold architecture. The freeze-drying process generates porosity through solvent sublimation, leading to irregular and poorly controlled pore networks, while 3D printing enables precise control over pore size, geometry, and interconnectivity [27,45,46,47,48]. The discrepancy between the large pores observed in the SEM micrographs and the pore size distributions obtained through image processing suggests that some macropores were segmented into smaller domains, resulting in an underestimation of their effective size. Nevertheless, both types of scaffolds exhibited hierarchical porosity that spanned multiple length scales.

From a biological perspective, the multiscale porosity observed in the 3D-printed scaffolds provides a plausible structural basis for the differences in cell viability and early osteogenic response. Macropores can facilitate fluid transport, cell infiltration, vascular ingrowth, and subsequent tissue penetration, while micro and submicron features increase the surface area for protein adsorption, cell attachment, and cell–material signaling [56,57,58]. In this context, the relatively open architecture of the 50% infill scaffold may explain its higher cell viability by improving nutrient and oxygen transport, while the more compact and uniformly microporous architecture of the 70% infill scaffold may promote greater cell–material contact and mechanotransduction associated with early osteogenic commitment. Conversely, the predominantly microporous and poorly interconnected architecture of the freeze-dried scaffolds may restrict transport and deep cellular infiltration. However, because the pore architecture was not manipulated independently of the fabrication method, infill density, and the formulation, these structure–function relationships should be interpreted as associations rather than direct causal mechanisms [56,57,58,59,60,61].

Compositional analysis revealed differences in the surface elemental profiles of the two scaffold types (Supplementary Figures S3 and S4). The freeze-dried scaffolds exhibited carbon-rich regions together with detectable calcium and phosphorus, while the analyzed region of the 3D-printed scaffold showed a stronger calcium signal but no detectable phosphorus. The higher calcium signal may indicate greater exposure of a calcium-containing inorganic component at the analyzed surface; however, in the absence of a corresponding phosphorus signal, it cannot be used alone to confirm a locally exposed HAp phase or a more homogeneous mineral distribution. Consequently, the possible relationship between surface calcium availability and the biological responses of 3D-printed scaffolds remains a hypothesis that should be examined by replicated EDX mapping and complementary compositional analyses [62,63,64,65].

The biological results are consistent with these structural and compositional observations. Quantitative analysis demonstrated significantly higher cell viability in 3D-printed scaffolds compared to freeze-dried scaffolds (*p* < 0.05), with the 50% infill group reaching approximately 85% viability. This result is likely associated with the presence of larger pores and improved interconnectivity, which facilitate nutrient diffusion, oxygen transport, metabolic waste removal, and cellular proliferation within three-dimensional biomaterials [56,57,58,59,60,61].

Interestingly, the scaffold exhibiting the highest cell viability was not the one with the strongest osteogenic response. While the 50% infill scaffold provides a more permissive environment for cell survival, the 70% infill scaffold demonstrated improved expression of early osteogenic markers (RUNX2 and OSX). This finding highlights a critical trade-off between porosity and biological function, where larger pores favor cell viability and transport phenomena, while more compact and refined architectures can promote cell–material interactions and osteogenic differentiation [56,57,58,59,60,61].

These results indicate that scaffold architecture influences not only cell survival but also lineage commitment, highlighting that favorable conditions for proliferation do not necessarily coincide with those that optimize osteogenic differentiation. On the contrary, freeze-dried scaffolds exhibited the lowest cell viability, with values close to 50% (Supplementary Figure S5). The observed differences in cell viability and osteogenic response are likely associated with the distinct microstructural features generated by each fabrication method. As discussed above, 3D-printed scaffolds exhibited a hierarchical and highly interconnected pore network, providing a more favorable microenvironment for cell survival, migration, and biological function [62,63,64,65,66,67,68,69,70].

The additive manufacturing process enables precise control over the scaffold architecture, allowing modulation of the pore size, interconnectivity, and permeability. These parameters directly influence the phenomena of mass transport, including nutrient diffusion, oxygen delivery, and metabolic waste removal, which are critical to maintaining cell viability in three-dimensional biomaterials [59,60]. Furthermore, a well-interconnected porous network facilitates cell migration and homogeneous distribution throughout the scaffold, promoting effective colonization and supporting tissue regeneration processes [60,61].

On the contrary, although the freeze-dried scaffolds exhibited a relatively rough surface that may enhance initial cell attachment, their lower effective porosity and limited pore interconnectivity likely restrict nutrient and oxygen transport to the scaffold interior. This limitation is consistent with the reduced cell viability observed in this group. Previous studies have demonstrated that insufficient pore interconnectivity significantly alters nutrient diffusion and metabolic waste, thus compromising cell proliferation, survival, and colonization within the inner regions of the scaffold [61]. These findings help explain why 3D-printed scaffolds, particularly those with optimized infill architectures, exhibited superior biological performance compared to their freeze-dried counterparts.

An additional formulation-related factor that should be considered is the presence of xanthan gum exclusively in the 3D-printing ink. Xanthan gum was incorporated as a rheological modifier to improve viscosity, flowability, and printability; however, it could also have influenced solvent retention, ice crystal formation during post-printing freeze-drying, pore morphology, interconnectivity, and the resulting mechanical and biological behavior. Because a xanthan-free 3D-printed control was not evaluated, the independent contribution of xanthan gum cannot be separated from the effects of printing, infill density, and scaffold architecture. Therefore, the differences between freeze-dried and 3D-printed scaffolds should be interpreted as resulting from combined fabrication- and formulation-dependent effects rather than from the fabrication method alone.

Consistent with the results of Alizarin Red S staining, which revealed increased mineralization-related activity in the 60% and especially 70% infill scaffolds, gene expression analysis further demonstrated a greater osteogenic commitment in these groups. All scaffolds supported the activation of osteogenic-related genes; however, the 3D-printed scaffold with 70% infill density exhibited the most favorable expression profile, with higher levels of RUNX2, OSX, ALP, and OPN compared to the other scaffold formulations. On the contrary, lower expression levels of these markers were observed in freeze-dried scaffolds and 3D-printed scaffolds with lower infill densities.

The elevated expression of RUNX2 and OSX in the 70% infill scaffold indicates an increased commitment of hDPSCs to the osteoblastic lineage, as these transcription factors play a central role in the initiation and progression of osteogenic differentiation [62,66,67,68,69,70]. Similarly, the expression of ALP in all groups confirms the onset of osteoblastic maturation and early mineralization processes, while the relatively low expression of COL1 suggests that extracellular matrix deposition remains in an early stage after 7 days of culture [62,63,64,65,66,67].

Increased expression of osteopontin (OPN), particularly in the 70% infill scaffold, further supports the activation of early mineralization-related pathways and cell–matrix interactions. Previous studies have shown that bioactive scaffolds can promote osteogenesis in the early stages by enhancing calcium deposition, protein adsorption, and osteoblastic differentiation [62,63,64,65]. In this context, the compositional differences observed by EDX analysis (Supplementary Figures S3 and S4), particularly the higher calcium availability in 3D-printed scaffolds, may contribute to the enhanced osteogenic response observed in these groups [68,69,70].

However, the biological findings should be interpreted within the temporal limits of this study. Live/dead staining, LDH analysis, osteogenic gene expression, and Alizarin Red S staining were performed after 7 days of culture and therefore characterize only early cell–material interactions and early osteogenic commitment. Although RUNX2, OSX, ALP, COL1, and OPN provide information on lineage commitment and initial matrix-related activity, late osteogenic markers such as osteocalcin and bone sialoprotein were not evaluated. Consequently, the present results do not demonstrate mature osteoblast differentiation or long-term mineralized matrix formation. Future studies should extend the evaluation to 14, 21, and 28 days and include late-stage osteogenic markers together with longitudinal viability, proliferation, and mineralization analyses.

Together, these findings suggest that the structural and compositional characteristics of the 70% infill scaffold provide a more favorable microenvironment for osteogenic differentiation. Combining a refined microporous network, adequate interconnectivity, and increased exposure of mineral phases can enhance cell–material interactions and mechanotransduction signaling, thus stimulating osteogenic gene expression [62,66,67,68,69,70]. Although lower infill densities favor cell viability through improved mass transport, higher infill densities appear to promote osteogenic commitment. This highlights a critical structure–function relationship, in which the scaffold architecture must be carefully optimized to balance cell survival and differentiation. In general, the 70% infill scaffold emerges as the most promising formulation to support early osteogenic commitment and bone tissue regeneration.

Degradation is a critical determinant of the clinical performance of PLGA/chitosan-based scaffolds because changes in scaffold mass, structure, local pH, and mechanical integrity may alter cell behavior, tissue ingrowth, and implant–scaffold stability. However, the present study did not include a standardized quantitative degradation assay. Dry mass loss, water uptake, molecular weight reduction, medium pH, released degradation products, and time-dependent retention of mechanical properties were not measured. Therefore, no conclusion regarding the degradation rate, onset of the degradation, or compatibility between scaffold degradation and new bone formation can be established from the current data. Future studies should conduct longitudinal degradation tests under physiologically relevant conditions and correlate mass loss, pH variation, polymer degradation products, morphological changes, and residual mechanical properties with cell viability and osteogenic maturation.

Although biological results highlight the regenerative potential of these scaffolds, their clinical applicability also depends on their ability to withstand physiological loading conditions. In this context, finite element analysis (FEA) performed using COMSOL Multiphysics^®^ provided valuable information on the biomechanical environment at the implant–scaffold interface [32,43]. Specifically, simulations were carried out using a 60% porous titanium implant (Brånemark™ System), allowing a rapid and cost-effective prediction of the behavior of the composite system under functional loading conditions. When considered alongside biological findings, these simulations contribute to a more comprehensive evaluation of scaffold performance by linking osteogenic potential with mechanical functionality. Recent computational studies of dental implantation have also evaluated porous scaffold configurations as alternatives to conventional fully dense implants. Hosseini and Katoozian et al. (2024) [71] compared fully porous and dense core porous scaffolds embedded in a jawbone model under static and dynamic masticatory loading. Their predictions of finite elements, supported by experimental strain gauge measurements, showed that the structure of the pores and geometric parameters substantially modify the stress distribution [71]. These findings support the use of FEA not only to evaluate the present implant–scaffold construct but also to guide systematic optimization of pore geometry, interfacial load transfer, and mechanical compatibility.

Mechanical benchmarking further clarifies the functional scope of the present scaffolds. The apparent stiffness values used in the FEA models ranged from 4 to 40 MPa, with values of 40 MPa for the highest-stiffness freeze-dried scaffold and 19 MPa for the highest-stiffness 3D-printed scaffold. These values are below the direction-dependent structural Young’s modulus reported for human vertebral cancellous bone, which ranges from approximately 43 to 165 MPa, and substantially below the tissue-level values reported for individual trabeculae and cortical bone, which are in the gigapascal range [41,72]. However, the present values are comparable to those of some highly porous polymer–ceramic scaffolds; for example, a PLGA/nHA scaffold containing chitosan microspheres exhibited a compressive modulus of approximately 29.4 MPa and a compressive strength of approximately 1.54 MPa [73]. Direct comparisons must be interpreted cautiously because mechanical values depend on scaffold composition, porosity, specimen dimensions, hydration state, loading mode, strain range, and whether structural or tissue-level properties are measured. Overall, this benchmarking indicates that the HAp/PLGA/CS scaffolds fall within the mechanical range of relatively compliant regenerative matrices, but remain substantially less stiff than the cancellous and cortical bone and therefore should not be considered load-bearing bone substitutes.

Consistent with this benchmarking, models 4 and 7 exhibited insufficient mechanical performance to reproduce the structural function of native cancellous bone, and their properties were markedly below those required to approximate cortical bone. Instead, they should be interpreted as bioactive matrices designed to support cell colonization, mechanotransduction, and osteogenesis during the early stages of regeneration. Despite its relatively low stiffness, the HAp/PLGA/CS system retains significant therapeutic potential by providing osteoconductive and osteoinductive signals that favor bone tissue formation [46,62,68,69,70].

FEA simulations further revealed that the mismatch between the elastic modulus of the titanium implant and that of the scaffold generated a nonuniform stress distribution at the interface. This mismatch may compromise primary stability and lead to localized implant subsidence under loading conditions. These findings are consistent with previous reports, such as those of Pérez et al. (2015) [43], which demonstrated that implants with controlled porosity can improve load transfer behavior and reduce the effects of stress shielding.

From a translational perspective, simultaneous implant placement with a PLGA-based regenerative construct has recently entered clinical evaluation. In a prospective single-arm study, Son et al. (2026) [74] used a resorbable PLGA membrane preformed in three dimensions for guided bone regeneration performed simultaneously with dental implant placement. After 5 months, the intervention produced a mean horizontal hard tissue gain of 2.64 mm and a hard tissue gain rate of approximately 87%, without exposure, infection, or wound dehiscence [74]. However, the device served as a space-maintaining barrier combined with a particulate graft rather than as a load-transmitting HAp/PLGA/CS scaffold. Consequently, this study supports the clinical feasibility of PLGA-based regenerative systems used simultaneously with implants, but does not provide direct clinical validation of the scaffold–implant configuration evaluated here.

Together, these results underscore the importance of achieving an optimal balance between porosity and mechanical strength. As demonstrated throughout this study, the scaffold architecture influences not only cell viability, osteogenic differentiation, and tissue regeneration but also mechanical performance and long-term functionality of the implant–scaffold system. Although the compressive properties measured experimentally fall within the lower range reported for porous bone, FEA simulations indicate that certain scaffold configurations may still be susceptible to excessive deformation under implant-related loading conditions. Therefore, further optimization of the scaffold design is required to enhance mechanical stability while preserving the favorable biological properties associated with hierarchical porosity and mineral phase distribution [34].

The translational implications of the present findings should be interpreted cautiously. The biological evaluation was performed under short-term in vitro conditions, and the finite element model represented a simplified mechanical scenario. Therefore, this study does not demonstrate in vivo bone formation, vascularization, osseointegration, long-term scaffold degradation, biological remodeling, or stable implant fixation. It also does not reproduce the complex cyclic, multidirectional, and time-dependent loading conditions of the oral environment. Consequently, observed cytocompatibility, early osteogenic response, and simulated mechanical behavior should be considered preclinical evidence supporting further investigation rather than evidence of clinical efficacy or readiness for simultaneous scaffold–implant placement.

Future studies should focus on the integration of optimized scaffold architectures with controlled delivery systems for osteogenic growth factors, as this combined approach may represent a promising strategy to further enhance cell viability, osteogenic differentiation, and regenerative outcomes in 3D-printed scaffolds. Although the 70% infill scaffold demonstrated potential to support early osteogenic commitment, further optimization and biological validation are required to fully determine its applicability for bone tissue regeneration. Furthermore, comprehensive investigations are needed to elucidate how variations in the density and architecture influence the mechanical behavior of the implant–scaffold interface. Computational approaches, particularly finite element analysis, should be used to identify scaffold designs capable of improving load transfer, minimizing mechanical mismatch, and achieving an optimal balance between structural integrity and biological functionality. These computationally optimized designs should subsequently be validated through experimental testing of the assembled implant–scaffold system under physiologically relevant static and cyclic loading conditions.

## 5. Conclusions

HAp/PLGA/CS scaffolds fabricated by freeze-drying and 3D printing exhibited excellent cytocompatibility with hDPSCs and supported early osteogenic differentiation. The 3D printing approach enabled precise architectural control, generating hierarchical and interconnected porous structures that improved cellular responses.

Scaffold architecture played a critical role in biological performance. The 50% infill scaffold promoted the highest cell viability, while the 70% infill scaffold induced the strongest osteogenic response, demonstrating that the pore architecture governs the balance between cell survival and osteogenic commitment.

Finite element analysis also showed that scaffold architecture influences the mechanical behavior of the implant–scaffold system, highlighting the need for additional mechanical optimization to improve load transfer while preserving biological performance.

Overall, the findings support the potential of architecturally controlled HAp/PLGA/CS scaffolds as experimental bioactive platforms for further bone tissue engineering research. However, because this study was limited to short-term in vitro evaluation and computational mechanical analysis, no conclusions can be drawn regarding bone regeneration, osseointegration, long-term degradation, biological remodeling, or implant fixation in vivo. Dedicated animal studies, longitudinal degradation analyses, cyclic mechanical testing, and experimental evaluation of implant stability will be required before the clinical relevance of the proposed scaffold–implant system can be established.

## Figures and Tables

**Table 1:** Primer sequences used for polymerase chain reaction (PCR).

Gene	Forward (5′-3′)	Reverse (3′-5′)
Runx2	CATCTAATGACACCACCAGGC	GCCTACAAAGGTGGGTTTGA
ALP	TCAGAAGCTCAACACCAACG	GTCAGGGACCTGGGCATT
OP	TGAAACGAGTCAGCTGGATGACCA	TGGCTGTGAAATTCATGGCTGTGG
OSX	TGGGAAAAGGGAGGGTAATC	CGGGACTCAACAACTCTGG
COL1	TGACCTCAAGATGTGCCACT	ACCAGACATGCCTCTTGTCC
GAPDH	GAAGGTGAAGGTCGGAGTC	GAAGATGGTGATGGGATTTC

**Table 2:** Freeze-drying scaffolds.

Compound *	Percentage %
Hydroxyapatite	1.07
PLGA	4.34
Chitosan	8.69
Chloroform	86.8

* The nominal mass ratio among the scaffold-forming components was HAp:PLGA:CS = 1:4:8.

**Table 3:** 3D-printing scaffolds.

Compound *	Percentage % **
Hydroxyapatite	1.89
PLGA	7.55
Chitosan	15.09
Xanthan gum ***	18.87

* The nominal mass ratio among the scaffold-forming components was HAp:PLGA:CS = 1:4:8. The minor deviation observed in the normalized ratio results from rounding of the reported formulation percentages. ** Percentage of the nonaqueous formulation. *** Xanthan gum and water were incorporated into the 3D-printing formulation to improve the flowability and printability of the material.

**Table 4:** Mechanical stiffness of scaffolds obtained by finite element analysis (FEA).

Model	Stiffness (MPa)	Scaffold Type (Fabrication Method)
4	40	Freeze-drying
7	19	3D printing
1	13	Freeze-drying
3	9	Freeze-drying
5	7	Freeze-drying
6	6	3D printing
2	4	Freeze-drying

**Table 5:** Mechanical properties of the scaffolds produced by the two methods.

Dimensions	Units	Mod. 1	Mod. 2	Mod. 3	Mod. 4	Mod. 5	Mod. 6	Mod. 7	Implant
Manufacturing	-	C. L.	C. L.	C. L.	C. L.	C. L.	3 D	3 D	-
Diameter	cm	0.78	0.80	0.78	0.78	0.80	0.78	0.70	0.33
Length	cm	1.73	1.09	1.06	1.91	1.48	1.03	1.00	0.80
Mass	g	2.75	2.96	3.07	2.89	2.94	3.18	1.85	0.30
Density	g/cm^3^	3.29	5.43	5.96	3.10	3.95	6.41	3.70	4.38
Modulus of elasticity	Mpa	13.0	4.0	9.0	40.0	7.0	6.0	19.8	10,000.0
Stress	Mpa	5.50	1.80	2.00	6.00	2.00	1.90	8.80	

## Data Availability

Data supporting the findings of this study are available from the corresponding author upon reasonable request. Raw and processed datasets include SEM image analysis, elemental composition data, mechanical testing results, RT-qPCR Ct values, Alizarin Red absorbance data, and finite element simulation outputs. The COMSOL model files and image analysis scripts can be made available upon request, subject to institutional data-sharing policies.

## Supplementary Materials

The following supporting information can be downloaded at https://www.mdpi.com/article/10.3390/biomimetics11080537/s1: Figure S1: SEM micrographs, depth maps, binary segmentations, and pore space segmentations of HAp/PLGA/CS scaffolds (A) freeze-dried and (B–D) 3D-printed at 50%, 60%, and 70% infill densities, respectively. Figure S2: Corresponding pore size distribution histograms (a–d) for the scaffolds shown in Figure S1. Figure S3: Energy-dispersive X-ray spectroscopy (EDX) analysis of HAp/PLGA/CS scaffolds fabricated via freeze-drying, showing (A) quantitative elemental composition and (B) representative EDX spectrum. Figure S4: Energy-dispersive X-ray spectroscopy (EDX) analysis of HAp/PLGA/CS scaffolds fabricated by 3D printing (60% infill), showing (A) quantitative elemental composition and (B) representative EDX spectrum. Figure S5: Cell viability of hDPSCs cultured on HAp/PLGA/CS scaffolds after 7 days of culture. Table S1: Summary of quantitative pore properties (fractional porosity, mean pore radius, standard deviation, and shape factor) of HAp/PLGA/CS scaffolds obtained by MATLAB-based image analysis, corresponding to samples A–D in Figures S1 and S2.

## Abbreviations

The following abbreviations are used in this manuscript:

HAp	Hydroxyapatite
PLGA	Poly (lactic-co-glycolic acid)
CS	Chitosan
hDPSCs	Human dental pulp stem cells
SEM	Scanning electron microscopy
EDX	Energy-dispersive X-ray spectroscopy
XRF	X-ray fluorescence
RT-qPCR	Reverse transcription quantitative polymerase chain reaction
ALP	Alkaline phosphatase
RUNX2	Runt-related transcription factor 2
OSX	Osterix
COL1	Type I collagen
OP	Osteopontin
FEA	Finite element analysis
FEM	Finite element method
