# Supplementary material for: HAp/PLGA/Chitosan Scaffolds Fabricated by Freeze-Drying and 3D Printing for Bone Regeneration: In Vitro Evaluation and Finite Element Analysis
Source: Biomimetics (Basel). 2026 Aug 2;11(8):537. doi: 10.3390/biomimetics11080537 (PMC13509448; doi:10.3390/biomimetics11080537)
Supplement: Supplementary file 1 [file biomimetics-11-00537-s001.zip › biomimetics-4417863-supplementary.pdf]

Supplementary Materials

Title of the manuscript: **HAp/PLGA/Chitosan Scaffolds Fabricated by Freeze-Drying and 3D Printing for Bone Regeneration: In Vitro Evaluation and Finite Element Analysis**

Manuscript ID: **biomimetics-4417863**

Figure S1

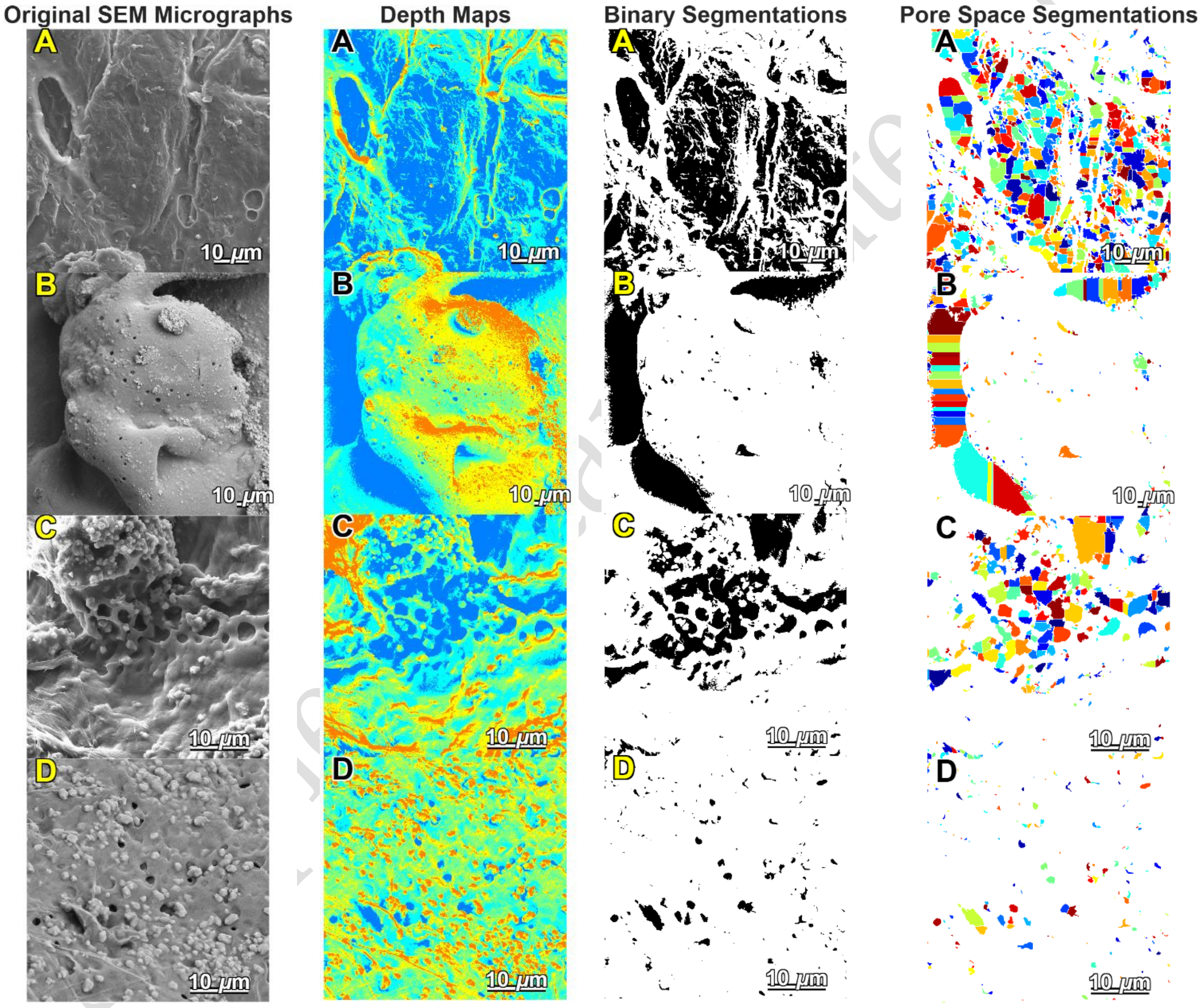

Figure S1. Original SEM micrographs, depth maps, binary segmentations, and pore-space segmentations of HAp/PLGA/CS scaffolds, obtained by MATLAB-based image analysis. (A) Freeze-dried scaffold. (B) 3D-printed scaffold, 50% infill density. (C) 3D-printed scaffold, 60% infill density. (D) 3D-printed scaffold, 70% infill density. Scale bars: 10 µm.

Figure S2

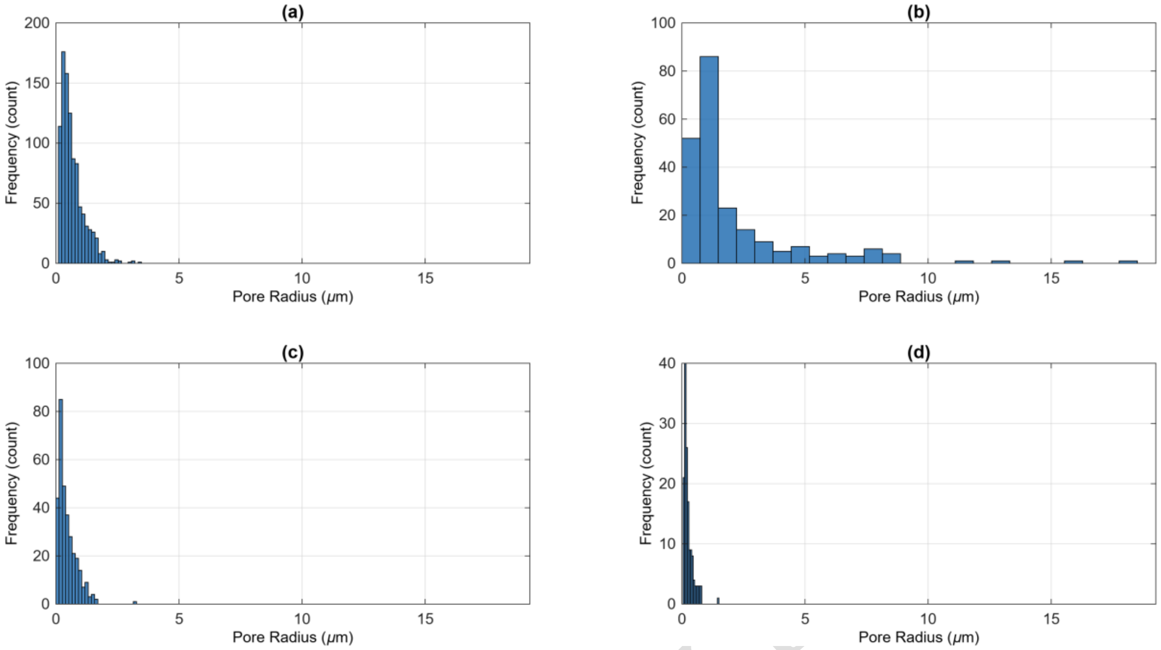

Figure S2. Pore-size distribution histograms (a: freeze-dried; b: 3D-printed 50% infill; c: 3D-printed 60% infill; d: 3D-printed 70% infill), corresponding to samples A–D shown in Figure S1.

Figure S3

Element	Wt%	Wt% Sigma	Atomic %
C	60.34	1.13	70.44
O	29.11	1.08	25.51
P	3.50	0.33	1.59
Ca	7.05	0.49	2.47
Total	100.0		100.0

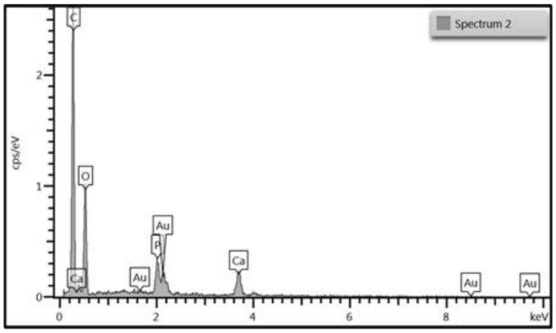

Figure S3. Energy-dispersive X-ray spectroscopy (EDX) analysis of HAp/PLGA/CS scaffolds fabricated via freeze-drying. (A) Quantitative elemental composition. (B) Representative EDX spectrum.

Supplementary

Figure S4

Element	Wt%	Wt% Sigma	Atomic %
C	21.73	1.01	30.54
O	57.60	1.03	60.76
Ca	20.66	0.69	8.70
Total:	100.0		100.00

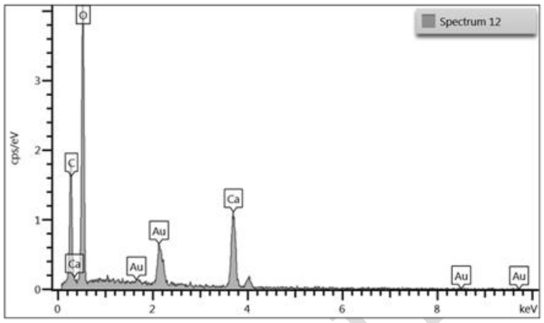

Figure S4. Energy-dispersive X-ray spectroscopy (EDX) analysis of HAp/PLGA/CS scaffolds fabricated via 3D printing (60% infill density). (A) Quantitative elemental composition. (B) Representative EDX spectrum. (Moved from the main manuscript, former Figure 7, in response to Reviewer 1.)

Figure S5.

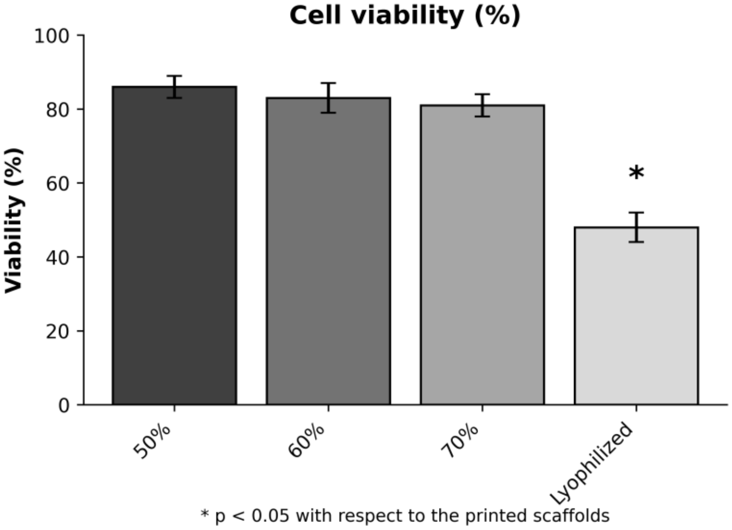

Figure S5. Cell viability of hDPSCs cultured on HAp/PLGA/CS scaffolds after 7 days of culture. The 3D-printed scaffolds showed higher cell viability than the lyophilized scaffold. The highest viability was observed in the 50% infill group. Data are presented as mean ± standard deviation. $p < 0.05$ with respect to the printed scaffolds.

Table S1

Table S1. Summary of quantitative pore properties of HAp/PLGA/CS scaffolds (samples A–D, corresponding to Figures S1 and S2), obtained by MATLAB-based SEM image analysis.

Table S1: Summary of Quantitative Properties

Sample	Fractional_Porosity	Mean_Radius_um	Std_Dev_um	Shape_Factor_SP
A	0.44605	0.67661	0.47448	0.0011531
B	0.19185	21.911	2.61	0.0062941
C	0.21947	0.4659	0.38399	0.0037055
D	0.029888	0.26185	0.1972	0.0095053

Supplementary material
